# Overcoming cancer-associated fibroblast-induced immunosuppression by anti-interleukin-6 receptor antibody

**DOI:** 10.1007/s00262-023-03378-7

**Published:** 2023-02-10

**Authors:** Noriyuki Nishiwaki, Kazuhiro Noma, Toshiaki Ohara, Tomoyoshi Kunitomo, Kento Kawasaki, Masaaki Akai, Teruki Kobayashi, Toru Narusaka, Hajime Kashima, Hiroaki Sato, Satoshi Komoto, Takuya Kato, Naoaki Maeda, Satoru Kikuchi, Shunsuke Tanabe, Hiroshi Tazawa, Yasuhiro Shirakawa, Toshiyoshi Fujiwara

**Affiliations:** 1 Department of Gastroenterological Surgery, Graduate School of Medicine, Dentistry and Pharmaceutical Sciences, Okayama University, 2-5-1 Shikata-Cho, Kita-ku, Okayama, 700-8558 Japan; 2 Department of Pathology & Experimental Medicine, Graduate School of Medicine, Dentistry and Pharmaceutical Sciences, Okayama University, Okayama, Japan; 3 Center for Innovative Clinical Medicine, Okayama University Hospital, Okayama, Japan; 4 Department of Surgery, Hiroshima City Hiroshima Citizens Hospital, Hiroshima, Japan

**Keywords:** Cancer-associated fibroblasts, Drug repositioning, Interleukin-6 receptor antibody, Tumor microenvironment

## Abstract

Cancer-associated fibroblasts (CAFs) are a critical component of the tumor microenvironment and play a central role in tumor progression. Previously, we reported that CAFs might induce tumor immunosuppression via interleukin-6 (IL-6) and promote tumor progression by blocking local IL-6 in the tumor microenvironment with neutralizing antibody. Here, we explore whether an anti-IL-6 receptor antibody could be used as systemic therapy to treat cancer, and further investigate the mechanisms by which IL-6 induces tumor immunosuppression. In clinical samples, IL-6 expression was significantly correlated with α-smooth muscle actin expression, and high IL-6 cases showed tumor immunosuppression. Multivariate analysis showed that IL-6 expression was an independent prognostic factor. In vitro, IL-6 contributed to cell proliferation and differentiation into CAFs. Moreover, IL-6 increased hypoxia-inducible factor 1α (HIF1α) expression and induced tumor immunosuppression by enhancing glucose uptake by cancer cells and competing for glucose with immune cells. MR16-1, a rodent analog of anti-IL-6 receptor antibody, overcame CAF-induced immunosuppression and suppressed tumor progression in immunocompetent murine cancer models by regulating HIF1α activation in vivo. The anti-IL-6 receptor antibody could be systemically employed to overcome tumor immunosuppression and improve patient survival with various cancers. Furthermore, the tumor immunosuppression was suggested to be induced by IL-6 via HIF1α activation.

**Supplementary Information:**

The online version contains supplementary material available at 10.1007/s00262-023-03378-7.

## Introduction

Cancer immunotherapy has led to breakthroughs in cancer treatment; however, the effects of immunotherapy are limited and have yet to overcome intractable cancers. Esophageal cancer (EC) is the seventh most common cancer and the sixth most common cause of cancer-related deaths globally [1]. Despite recent advances in EC-associated chemotherapy, targeted therapy, and immunotherapy, the prognosis remains poor with a 5-year survival rate of approximately 15–25% [2, 3]. Moreover, preclinical or clinical studies consistently report mixed results, which suggests that the tumor microenvironment (TME), especially the immune microenvironment in EC, may be implicated in the regulation of those therapies [4, 5].

Numerous studies have demonstrated that the TME composition significantly influences tumor outcomes [6–8]. Cancer-associated fibroblasts (CAFs) are critical components of the TME and play a central role in tumor growth, metastasis, and invasion [9, 10], furthermore have recently attracted attention as potential therapeutic targets [10–12]. In EC patients specifically, CAFs contribute to tumor development by promoting angiogenesis [13], chemoresistance [14], lymph node metastasis [10], and tumor immunosuppression [15]. Previously, we reported that CAF elimination suppresses tumor growth [16] and neutralizing local Interleukin-6 (IL-6) in the TME secreted by CAFs improves tumor immunosuppression [15].

Although it is widely known that CAFs are central players in shaping the TME toward immunosuppression by mediating the immune system [17], we focused on the IL-6-mediated recruitment of tumor-infiltrating immune cells by CAFs and their fate in a hypoxic TME. Most solid tumor regions are permanently, or transiently, hypoxic due to aberrant vascularization and poor blood supply [18]. Hypoxic environments and subsequent activation of hypoxia-inducible factor 1α (HIF1α) are common features of advanced cancers. Under hypoxic conditions, HIF activity contributes to increased tumor glycolysis, causing “metabolic competition” between cancer cells and T-cells, while suppressing T-cell function and the antitumor response [19]. Although we reported that CAFs alter T-cell distribution in the TME to an immunosuppressive state via IL-6 [15], the precise mechanism is not yet clear.

Tocilizumab (TCZ) is the first marketed IL-6 blocking antibody that targets IL-6 receptors and has been used to treat rheumatoid arthritis [20]. Although the tumor growth effect of IL-6 is well-known and the application of anti-IL-6 receptor antibodies to cancer treatment has been attempted, there are few reports showing clear therapeutic effects [21–23]. We hypothesized that IL-6 produced by CAFs promotes tumor growth in the TME and is the target of anti-IL-6 receptor antibody therapy. Specifically, we aimed to determine whether anti-IL-6 receptor antibody overcomes tumor immunosuppression and suppresses tumor progression using systemic administration of MR16-1, which is a rodent analog of TCZ [24]. Further, we explored the mechanism by which CAFs induce immunosuppression via IL-6, especially focused on hypoxic TME.

## Materials and methods

### Patients and clinical information

A total of 185 EC tumor samples were obtained from patients who underwent esophagectomy with lymph node dissection at Okayama University Hospital between 2008 and 2011. The outline of our study was published on our web page to explain the study and to provide opportunities for disagreement. Surgeries were performed according to the Japanese EC treatment guidelines [25, 26]. Patients were excluded if they: (i) underwent follow-up procedures; (ii) were diagnosed with melanoma or distant metastases; or (iii) were in complete remission. Resected specimens were fixed with 10% formalin. Tumor classification and stage were determined according to the TNM Classification of Malignant Tumors 7th edition (UICC 7th edition) [27].

### Reagents and antibodies

A rat anti-mouse-IL-6 receptor antibody, MR16-1, was kindly provided by Chugai Pharmaceutical Co., Ltd. (Tokyo, Japan). Details of the other reagents and antibodies used in this study are listed in Table S1.

### Cell lines

Murine colon cancer (Colon26), murine fibroblast (NIH/3T3), human esophageal squamous cell cancer (TE4), and human esophageal adenocarcinoma (OE19) cell lines were purchased from the Japanese Collection of Research Bioresources (JCRB, Osaka, Japan) Cell Bank. Murine fibroblast cell line (MEF) was purchased from the American Type Culture Collection (ATCC, Manassas, VA, USA). Murine squamous cell carcinoma cell line (SCCVII) was kindly provided by Professor Yuta Shibamoto (Nagoya City University, Nagoya, Japan), and murine pancreatic ductal adenocarcinoma (Pan02) was obtained from the National Cancer Institute (Frederick, MD, USA). Primary human esophageal fibroblasts (FEF3) were isolated from the human fetal esophagus as described previously [13]. WI-38 fetal lung human fibroblasts were purchased from the Health Science Research Resource Bank (Osaka, Japan).

### Immunohistochemistry (IHC)

All IHC procedures were described previously [15]. Stained slides were evaluated using ImageJ software (http://rsb.info.nih.gov/ij/). Briefly, the number of cells expressed with CD8, Forkhead box protein 3 (FoxP3), ionized calcium-binding adaptor protein 1 (Iba1), CD163, and HIF1α were counted in four randomly selected high-magnification fields. The scores of alpha smooth muscle actin (αSMA), IL-6, and vascular endothelial growth factor (VEGF) were evaluated using an “area index,” calculated in low magnification fields. All evaluations were performed by an independent pathologist who was blinded to clinical information.

### Immunofluorescence (IF)

Primary antibodies were added to deparaffinized slides for 60 min at room temperature (RT) (20–22 °C) or overnight at 4 °C, followed by secondary antibodies for 60 min at RT. Coverslips were coated with a drop of mounting medium (P36983; Invitrogen, Thermo Fisher Scientific, Waltham, MA, USA) and subsequently photographed using a fluorescence microscope (IX83; Olympus, Tokyo, Japan).

### Cell viability assay

Cells were seeded in 96-well plates (10 × 10^4^ cells/well) and treated with recombinant IL-6 and recombinant IL-6 receptor alpha (IL-6Rα). According to the manufacturer’s protocol, cell viability was determined 2 days after treatment using a Cell Proliferation Kit II (XTT; Roche Diagnostics, Rotkreuz, Switzerland).

### ELISA

Cell culture supernatants and human serum samples were assessed for the levels of mouse-IL-6, mouse-IL-6Rα, and human-IL-6 using appropriate ELISA kits (R & D Systems), according to the manufacturer’s protocol.

### Western blot analysis

Proteins were extracted from whole-cell lysates or nuclear proteins, electrophoresed on polyacrylamide gels and transferred onto membranes. The membranes were incubated with primary antibodies overnight at 4 °C, followed by secondary antibodies 60 min at RT, and then visualized using the Amersham ECL chemiluminescence system (GE Healthcare, IL, USA). Equal loading of the samples was confirmed using β-actin.

### Animal studies

Animals were maintained under specific pathogen-free conditions at the Department of Animal Laboratory at Okayama University. Mice were purchased from Clea (Tokyo, Japan) and housed under sterile conditions.

### Subcutaneous syngeneic cancer mouse model

Colon26 (0.5 × 10^6^) cells with and without NIH/3T3 (0.5 × 10^6^) cells were subcutaneously inoculated into the right flank of 6-week-old female BALB/c mice. The perpendicular diameter of each tumor was measured every 3 days. Tumor volume was calculated using the formula:

$${\text{Tumor volume }}\left( {{\text{mm}}^{{3}} } \right) \, = \, L \, \times \, W^{2} \times \, 0.5$$

*L* represents the longest diameter, *W* represents the shortest diameter, and 0.5 is a constant used to calculate the volume of an ellipsoid. Treatment with intraperitoneal injections of 20 mg/kg of isotype control (BE0088; BioXcell, Lebanon, NH, USA) or MR16-1 every 3 days began when tumors reached 50–100 mm^3^. To generate other cancer models, Pan02 and MEF models were established in C57BL/6 mice, while SCCVII and MEF models were established in C3H/He mice, which were then inoculated and treated in the same way as the Colon26 model.

For T-cell depletion studies, anti-CD8α antibodies (BP0061; BioXcell) were injected intraperitoneally at 10 mg/kg per day before the first injection of isotype control or MR16-1, and every 3 days thereafter, for a total of four treatments. The animals were euthanized via cervical dislocation, and serum and tumor tissue were collected for further analyses.

### Culture of mouse bone marrow-derived monocytes

Mouse bone marrow-derived monocytes (BMDMs) were isolated from the femur bones of 6- to 10-week-old BALB/c female mice according to the previous studies [28–30]. BMDMs were used as a positive control for macrophage differentiation experiments using IL-4 or IL-6 as stimuli [29].

### Isolation of tumor-infiltrating lymphocytes (TILs)

Tumor tissues were dissected from the mice, and TILs were harvested using BD Horizon Dri Tumor and Tissue Dissociation Reagent (TTDR), according to the manufacturer’s protocol. All cells, including TILs and tumor cells with indicated fluorescence-labeled antibodies, were subjected to flow-cytometric analysis.

### Flow-cytometric analysis

Cells were washed and incubated with monoclonal antibodies for 30 min at RT in PBS containing 2% FBS. Cells were then washed and analyzed on a BD FACSAria III or FACSLyric (BD Biosciences).

### Intracellular cytokine staining of TILs

TILs were harvested as described above and stimulated for 6 h in the presence of phorbol-12-myristate-13-acetate (PMA), ionomycin, and Brefeldin A at 37 °C. Next, cells were harvested and labeled with a cell surface marker followed by intracellular cytokine staining and flow-cytometric analysis on a FACSAria III.

### Statistics

All statistical analyses were performed using JMP software (SAS Institute, Cary, NC, USA). Overall survival (OS) and disease-free survival (DFS) were calculated using the Kaplan–Meier method, with the log-rank test to compare subgroups. Hazard ratios (HRs) and 95% confidence intervals (CIs) for clinical variables were calculated using Cox proportional hazard regression in univariate and multivariate analyses. Spearman’s correlation was used to assess relationships between variables. For group comparisons, the Mann–Whitney test or Student’s *t* test was used. For multiple-group comparisons, analysis of variance with Tukey’s test was used. Statistical significance was set at *P* < 0.05.

### Study approval

This study was conducted in accordance with the Declaration of Helsinki’s ethical standards and the ethical guidelines for medical and health research involving human subjects. The use of clinical samples was approved and reviewed by the Ethics Review Board of Okayama University (No. 1801-023; Okayama, Japan). The experimental animal protocol was approved and reviewed by the Ethics Review Committee for Animal Experiments at Okayama University (OKU-2020166).

## Results

### IL-6 expression is an independent prognostic factor in EC patients

**Fig. 1:**
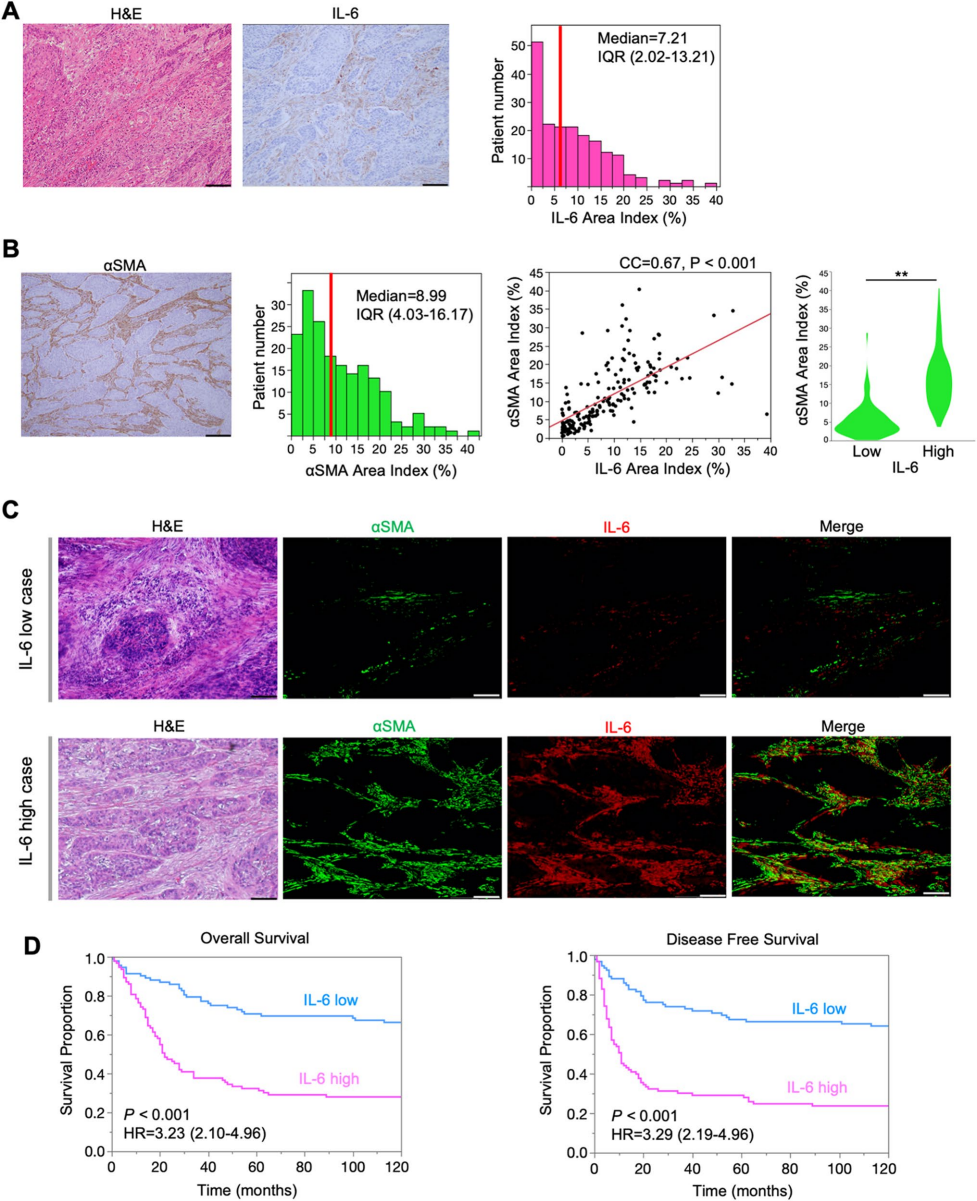
Correlation of IL-6 expression and CAFs distribution in esophageal cancer tissues. **A** Tissue staining with H&E and IL-6. ImageJ was used to evaluate the area index at 200 × magnification. The IL-6 area index is plotted as a histogram (red bar, median value). Scale bars: 100 µm. **B** The area index of αSMA at 100× magnification was recorded using ImageJ. Scale bars: 200 µm. Correlation between IL-6 and CAFs is shown by the scatter plot (Spearman’s correlation coefficient). Violin plots show comparisons based on high or low IL-6 area index. ^**^P < 0.01, Student’s *t* test. **C** IF images of IL-6 and αSMA. Representative high and low IL-6 cases at 100× magnification. Scale bars: 200 µm. **D** Survival curve according to the IL-6 expression (low or high group). Cox regression hazard model, 95% confidence intervals, and log-rank test.

We conducted IL-6 IHC analysis of surgically resected specimens, and the mean value was calculated as an “IL-6 area index” (Fig. 1A). The expression of IL-6 was significantly correlated with the expression of αSMA (*r* = 0.67, *P* < 0.001) (Fig. 1B). When patients were divided into high and low IL-6 groups based on the median value (7.21) of IL-6 area index, IF imaging revealed that the expression of αSMA (green) and IL-6 (red) overlapped in both high and low CAF groups (Fig. 1C). We evaluated the relationship between IL-6 expression, clinicopathological features, and clinical outcomes in 185 patients with EC (Supplementary Table 2). Univariate analysis revealed that sex, tumor depth, lymph node status, prior neoadjuvant therapy administration, αSMA and IL-6 expression, CD8^+^ (cytotoxic T-cells (CTL)) and FoxP3^+^ (regulatory T-cells (Treg)) TILs status, and CD163^+^ tumor-associated macrophages (TAMs; M2 macrophages) status were significant prognostic factors for OS (Table 1). Patients with high IL-6 expression had significantly shorter OS and DFS than those with low expression (Fig. 1D). OS stage-related subgroup analysis using intratumoral tissues revealed that lower IL-6 expression tended to reflect a better OS for all stages (Fig. S1). Multivariate analysis was performed using all variables via univariate analysis with *P* < 0.10; a backward selection was performed using the Akaike information criterion. Multivariate analysis identified IL-6 expression as an independent prognostic factor for OS (HR = 1.82, 95% CI = 1.03–3.20, *P* = 0.039: Table 1). Similar trends were observed for DFS (Table S3).

**Table 1:** Univariate and multivariate analysis for overall survival

		Univariate analysis			Multivariate analysis		
Variable	Unfavorable/favorable	HR	95%CI	*P* value	HR	95%CI	*P* value
Age (years)	> 66/ ≤ 66	1.36	0.91–2.02	0.131			
Sex	Male/Female	2.98	1.21–7.32	0.017*			
Histological type	Adenocarcinoma/SCC	0.72	0.33–1.56	0.404			
Pathological T stage	T3-4/T1-2	3.68	2.41–5.61	< 0.001*	2.48	1.43–4.30	0.001*
Pathological N stage	N1-3/N0	2.59	1.70–3.98	< 0.001*	1.55	0.96–2.53	0.075
Neoadjuvant therapy	yes/no	2.10	1.37–3.22	< 0.001*			
αSMA	high/low	2.99	1.95–4.57	< 0.001*			
IL-6	high/low	3.23	2.10–4.96	< 0.001*	1.82	1.03–3.20	0.039*
Tumor-infiltrating lymphocytes							
CD8 +	high/low	0.59	0.39–0.88	0.009*	0.72	0.47–1.10	0.124
FoxP3 +	high/low	2.40	1.58–3.63	< 0.001*			
Tumor-associated macrophages							
Iba1 +	high/low	1.09	0.74–1.63	0.658			
CD163 +	high/low	1.49	1.00–2.22	0.050	0.64	0.40–1.00	0.051

Cox proportional hazard model, Statistical significance: ^*^P value < 0.05, HR: hazard ratio; CI: confidence interval; SCC: squamous cell carcinoma; SMA: smooth muscle actin; IL-6: interleukin-6; FoxP3: Forkhead box p3; Iba1: ionized calcium-binding adaptor protein1

### CAFs induce immunosuppression via IL-6 in the TME

**Fig. 2:**
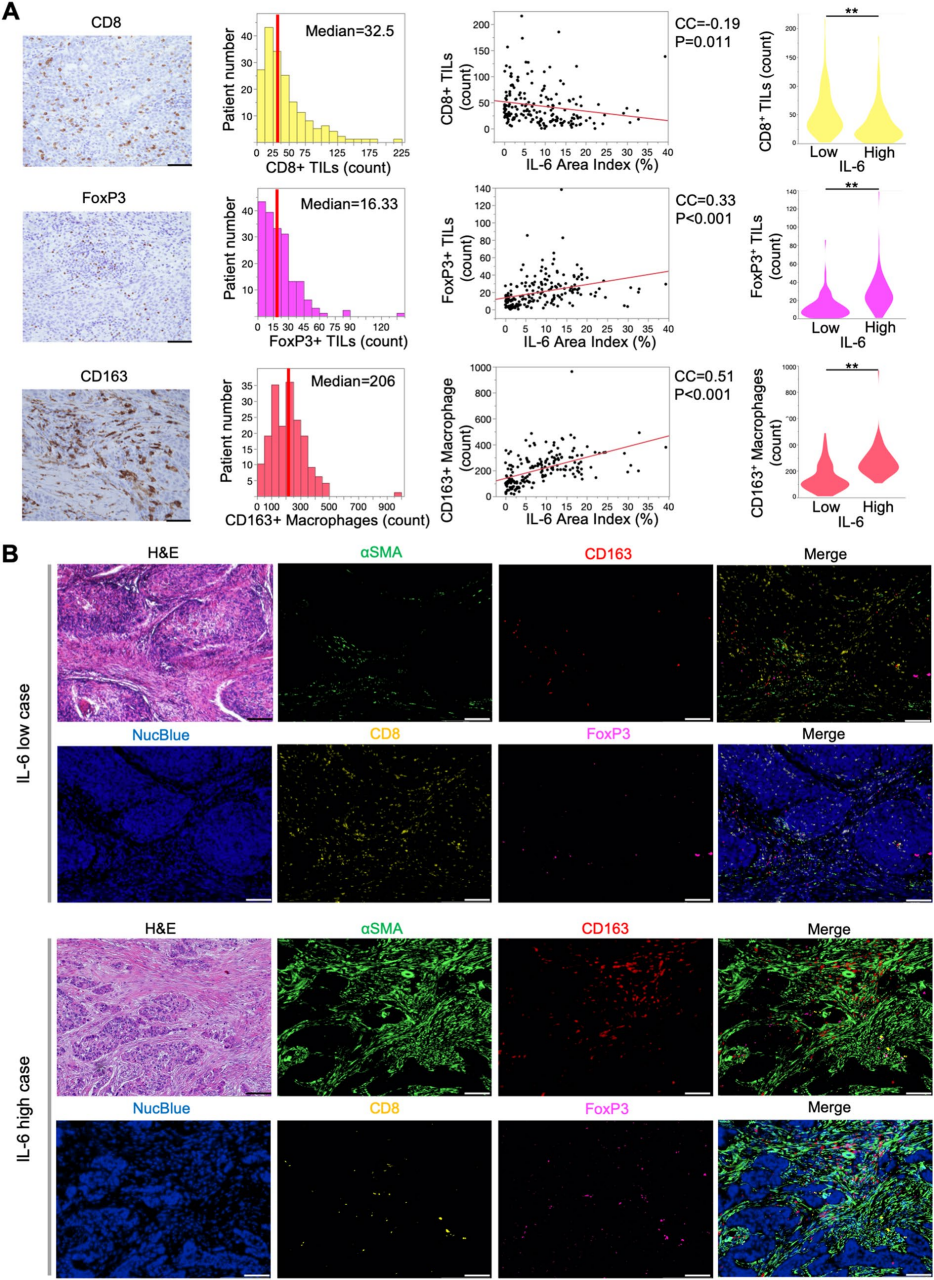
Relative distribution of CAFs, IL-6, and immune cells in resected human esophageal cancer samples. **A** Average number of CD8^+^ or FoxP3^+^ TILs, and Iba1^+^ or CD163^+^ TAMs at 400× magnification. Scale bars: 50 µm. Correlation between IL-6 and CD8^+^ or FoxP3^+^ TILs, CD163^+^ TAMs is shown by scatter plot (Spearman’s correlation coefficient). Violin plots show comparisons based on high or low IL-6 area index. ^**^P < 0.01, Student’s *t* test. **B** IF images of CD8- or FoxP3-expressing lymphocytes, CD163-expressing macrophages, and αSMA. An example of high and low IL-6 cases at 100× magnification. Scale bars: 200 µm

In intratumoral tissues, negative correlations between CD8^+^ TILs and IL-6 (*r* = − 0.19), and positive correlations between IL-6 and FoxP3^+^ TILs (*r* = 0.33), and CD163^+^ TAMs (*r* = 0.51; Fig. 2A), were observed. In a comparison based on the IL-6 area index, those with a high IL-6 area index showed significantly lower CD8^+^ with higher FoxP3^+^ TIL and CD163^+^ TAM quantities in intratumoral tissues. In contrast, no significant correlation was observed between Iba1^+^ TAM (pan-macrophage) numbers and IL-6 (Fig. S2).

IF imaging revealed that CD8^+^ TILs were scarce in high IL-6 patients, despite αSMA accumulation. Unlike CD8^+^ TILs, the abundance of FoxP3^+^ TILs and CD163^+^ TAMs increased in the high IL-6 group compared to the low IL-6 group (Fig. 2B).

### IL-6 directly contributes to cancer and stromal cell proliferation and differentiation into CAFs and TAMs

**Fig. 3:**
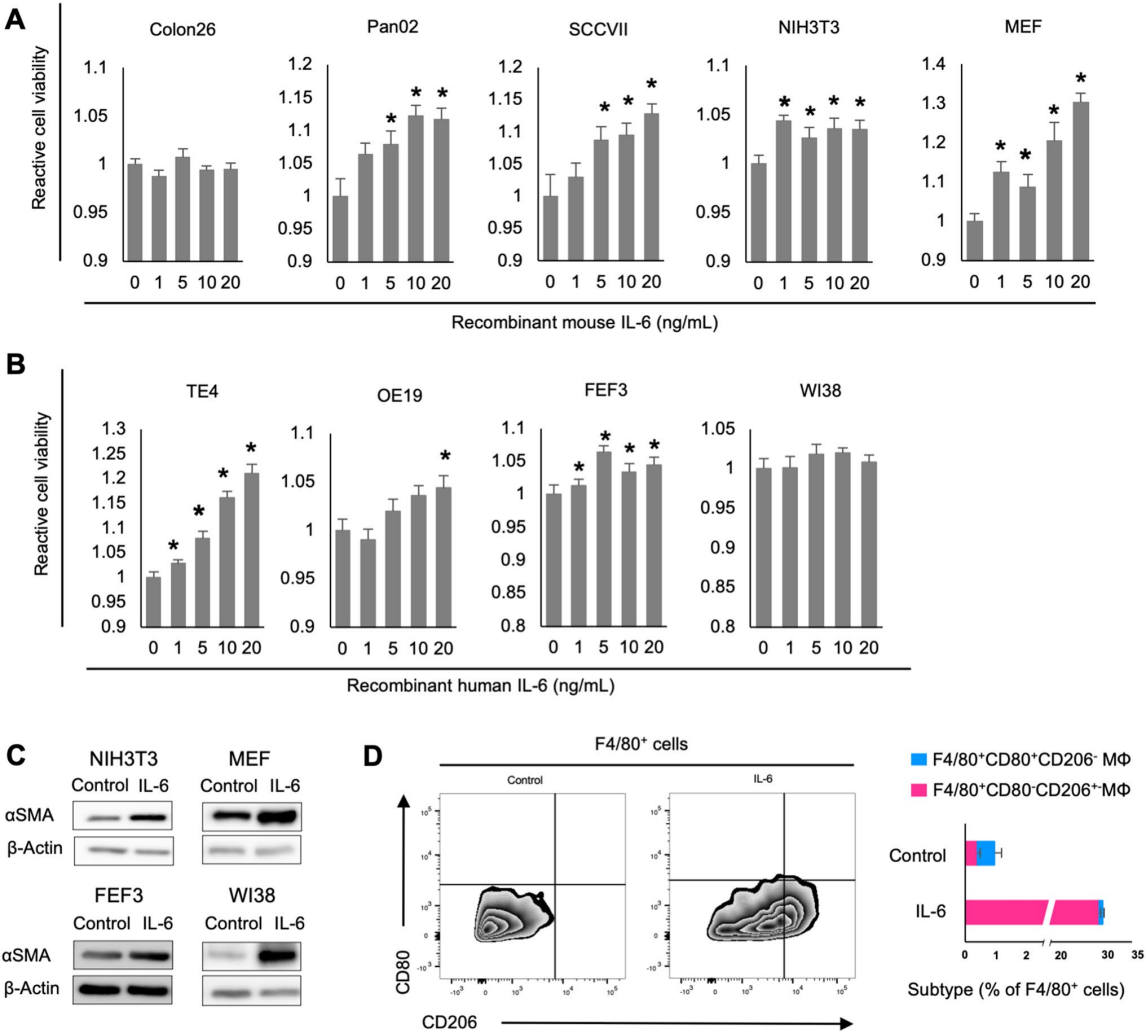
Direct contribution of IL-6 to cell proliferation and differentiation of CAFs and TAMs. **A** and **B** Percentage of viable cells at various concentrations of recombinant IL-6 (1, 5, 10, 20 ng/mL) relative to untreated cells (0 ng/mL). Recombinant IL-6R alpha was administered at five times the IL-6 concentration; *n* = 5; mean ± SE. ^*^P < 0.05, Student’s *t* test compared to untreated control. **A** Murine cell lines. **B** Human cell lines. **C** Whole-cell lysates of NIH/3T3, MEF, and FEF3 cells collected 2 days after IL-6 treatment (20 ng/mL) subjected to western blot analysis of αSMA and β-actin expression. **C** Flow cytometry analysis of cell surface F4/80 (M1/M2 marker) and CD80 (M1 marker), and intracellular CD206 (M2 marker) expression in BMDMs with or without IL-6 (20 ng/mL) treatment for 2 days. The bar chart shows the quantification of the F4/80^+^, CD80^+^, and CD206^−^ (M1) population and F4/80^+^, CD80^−^, and CD206^+^ (M2) populations, *n* = 3

An XTT assay was conducted to evaluate cell proliferation. IL-6-induced proliferation of both cancers and fibroblasts for murine and human cell lines; no difference was observed in Colon26 and WI38 cells (Fig. 3A). Western blot analysis revealed that IL-6 treatment increased the expression of αSMA; thus, IL-6 differentiated normal fibroblasts into CAFs (Fig. 3C).

The effect of IL-6 on macrophage polarization was investigated using BMDMs that were primed for differentiation and pretreated with mouse macrophage colony-stimulating factor (M-CSF). Flow cytometry analysis of F4/80 (pan-macrophage), CD80 (M1 marker), and CD206 (M2 marker) expression showed that compared with control cells, treatment with IL-6 increased differentiation of F4/80^+^CD80^−^CD206^+^ macrophages, indicating an M2-like phenotype (Fig. 3D).

### MR16-1 overcomes tumor immunosuppression and suppress tumor growth in vivo

**Fig. 4:**
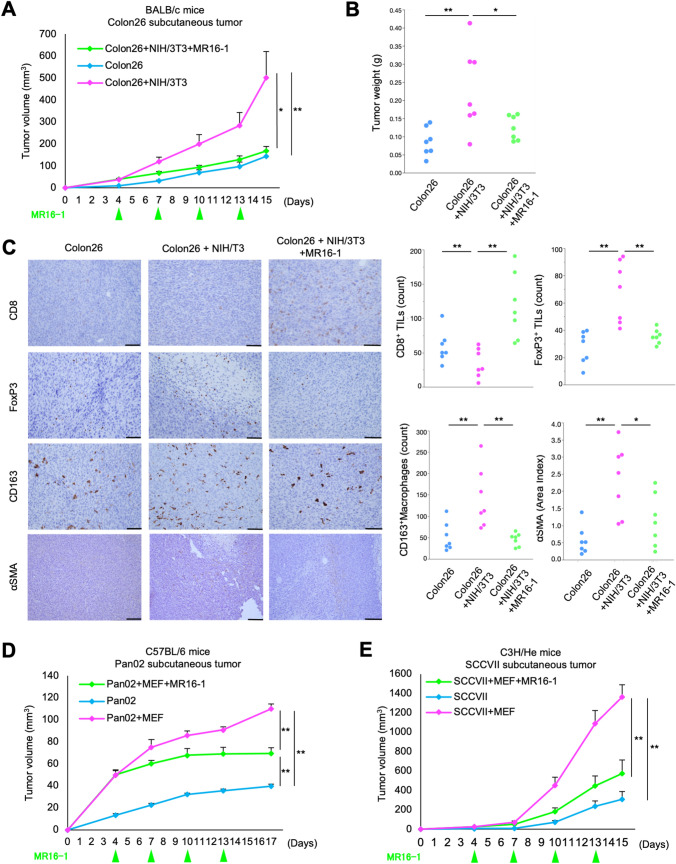
MR16-1 treatment overcomes tumor immunosuppression and suppresses tumor growth. **A** Volume and **B** weight of tumors excised from Colon26 + NIH/3T3 mice, *n* = 7 mice/group; mean ± SE. ^*^P < 0.05; ^**^P < 0.01, Tukey's test with ANOVA. **C** IHC staining for CD8, FoxP3, CD163, and αSMA in tumor tissues. The average number of CD8^+^ or FoxP3^+^ TILs and CD163^+^ TAMs at 400× magnification and the area index of αSMA at 200× magnification. Scale bars: 100 µm (200 ×); 50 µm (400 ×). ^*^P < 0.05; ^**^P < 0.01, Tukey's test with ANOVA. **D** and **E** Tumor volume of the transplanted mice in each group. **D** Pan02 + MEF model, *n* = 5 mice/group, **E** SCCVII + MEF model, *n* = 5 mice/group; mean ± SE. ^*^P < 0.05; ^**^P < 0.01, Tukey's test with ANOVA

Previously, we demonstrated that CAFs contribute to tumor growth by inducing tumor immunosuppression via IL-6 using in vivo experimental models [15]. To evaluate the effect of MR16-1, a TCZ analog for tumor suppression, we performed in vivo experiments using Colon26 cells and BALB/c mice. Tumors that developed through inoculation with cancer cells (Colon26), co-inoculation with fibroblasts (Colon26 + NIH/3T3), or co-inoculation and treatment with MR16-1 (Colon26 + NIH/3T3 + MR16-1) were compared. MR16-1 significantly reduced the accelerated growth (Fig. 4A) and tumor weights (Figs. 4B and S3A) that were observed in the co-inoculated tumors. IHC demonstrated that the number of CD8^+^ TILs in the Colon26 + NIH3T3 group was lower than in the Colon26 group. In contrast, an increased proportion of FoxP3^+^ TILs and CD163^+^ TAMs were observed in the Colon26 + NIH3T3 group compared to the Colon26 group (Fig. 4C). No difference was observed in the number of Iba1^+^ TAMs (Fig. S3B). Notably, MR16-1 influenced the TIL and TAM populations in the TME, with a significant increase in CD8^+^ TILs and a significant decrease in FoxP3^+^ TILs and CD163^+^ TAMs, compared with the Colon26 + NIH3T3 group. IHC revealed that the expression of αSMA was higher in the Colon26 + NIH3T3 group than in the Colon26 group and decreased in the MR16-1 group (Fig. 4C).

We performed the same experiment, substituting Colon26 cells for the pancreatic cancer cell line Pan02 in C57BL/6 mice (Figs. 4D and S4). The same trends of tumor suppression and overcoming immunosuppression by MR16-1 were observed as for Colon26 tumors. We conducted the same study with a dermal squamous cell carcinoma cell line SCCVII in C3H/He mice to mimic esophageal squamous cell cancer (Figs. 4E and S5). The same trends were observed. Importantly, MR16-1 treatment did not induce significant weight loss in any of the animals tested (Figs. S3D, S4C, S5C).

### MR16-1 suppresses tumor growth by affecting CD8^+^ T cells in the TME in vivo

**Fig. 5:**
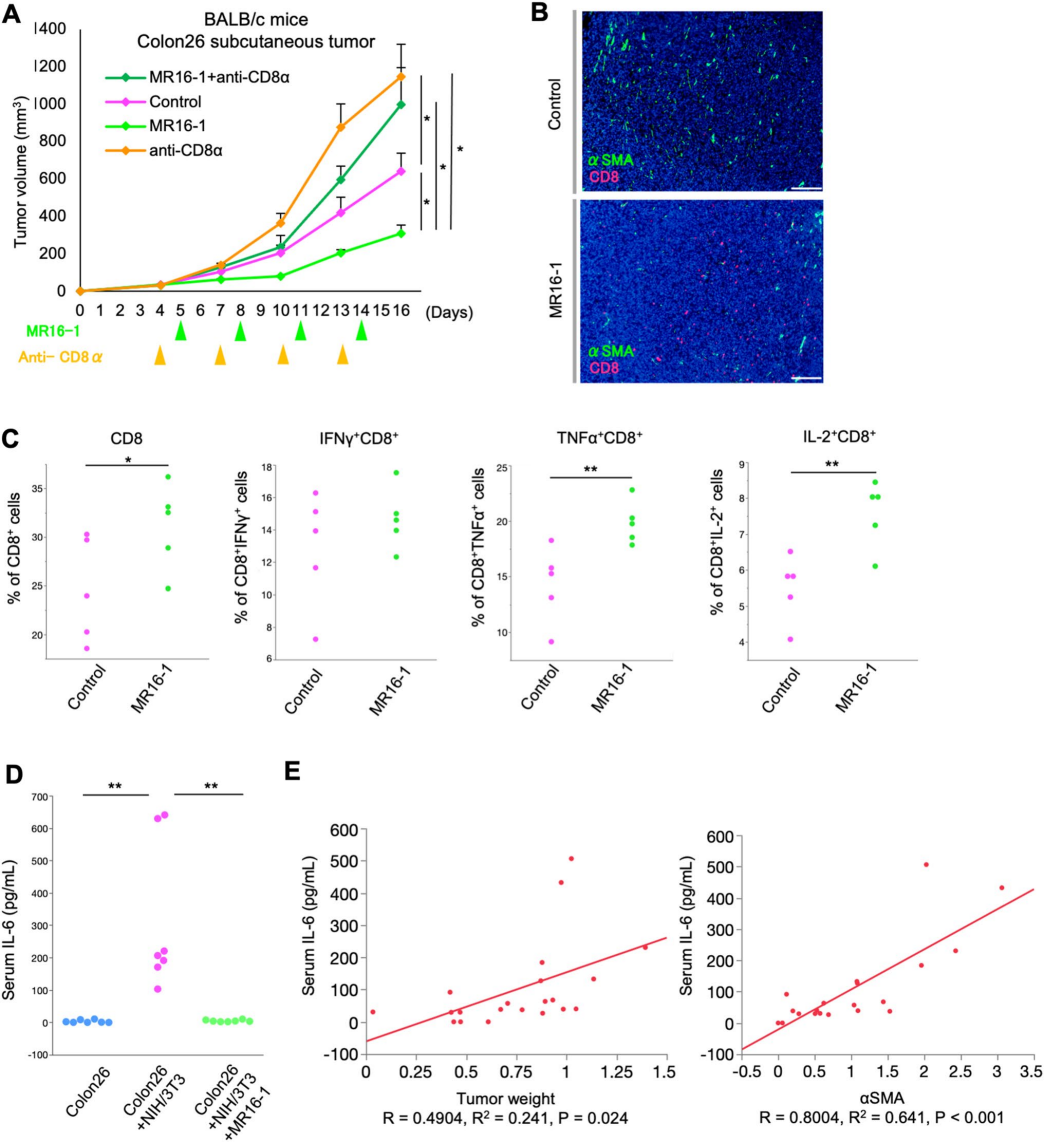
MR16-1 suppresses tumor growth by affecting CD8^+^ T cells. **A** Tumor volume in transplanted mice with or without MR16-1 treatment. Mice depleted of CD8^+^ T-cells; *n* = 5 mice/group mean ± SE. ^*^, *P* < 0.05, Tukey’s test with ANOVA. **B** IF images of αSMA and CD8-expressing lymphocytes in control and MR16-1 treatment groups at 100× magnification. Scale bars: 200 µm. **C** Colon26 + NIH/3T3 tumors treated with or without MR16-1 analyzed for TILs via flow cytometry. TILs were stimulated with PMA/ionomycin for 6 h, stained for surface CD8, and intracellular staining for IFNγ, TNFα, and IL-2. Flow-cytometric analyses of cytokine-producing CD8^+^ TILs were statistically assessed between control and MR16-1 treatment (*n* = 5). ^*^P < 0.05; ^**^P < 0.01, Student’s *t* test. **D** Serum IL-6 quantification in Colon26 + NIH/3T3 model by ELISA. ^**^P < 0.01, Tukey’s test with ANOVA. **E** Correlation between serum IL-6 and tumor weight or αSMA (Spearman’s correlation coefficient)

We hypothesized that MR16-1 suppressed tumor progression by increasing and activating CD8^+^ TIL and evaluated whether the efficacy of MR16-1 was CD8^+^ TIL dependent. Colon26 + NIH/3T3 mice were administered the CD8α depleting antibody during treatment. Four fibroblast groups co-inoculated with cancer cells were compared: no treatment (control), treated with MR16-1 (MR16-1), treated with anti-CD8α antibody (anti-CD8α), and treated with MR16-1 and anti-CD8α (MR16-1 + anti-CD8α). Administration of CD8α depleting antibody abrogated the efficacy of MR16-1 in mice bearing Colon26 + NIH/3T3 tumors (Figs. 5A and S6). Tumor progression was significantly suppressed in the MR16-1 group compared to the control and combined MR16-1 and CD8α depleting antibody groups.

We investigated the status of CD8^+^ TILs in the control and MR16-1 groups. IF staining and flow cytometry demonstrated that the number of total CD8^+^ TILs in the MR16-1 group was higher than the controls (Fig. 5B, C). Furthermore, the capacity for triple cytokine production of CD8^+^ TILs was increased by MR16-1 treatment (IFNγ, *P* = 0.170; TNFα, *P* = 0.007; IL-2 *P* = 0.004).

### Serum IL-6 may serve as a biomarker of CAFs in the TME

Serum samples from mice were analyzed for IL-6 and IL-6Rα. Serum IL-6 concentration in the Colon26 + NIH3T3 group was higher than the Colon26 group, while MR16-1 treatment decreased IL-6 (Fig. 5D). In contrast, IL-6Rα was the highest in the MR16-1 group (Fig. S3C). To investigate the relationship between CAFs in the TME and serum IL-6, we analyzed three groups with varying amounts of fibroblasts: cancer cells alone (Colon26), co-inoculated cancer cells and fibroblasts (Colon26 + 1NIH/3T3, 1:1), and co-inoculated cells with Colon26 + 2NIH/3T3 (1:2). The protocol was followed by tumor resection and simultaneous blood sampling once the tumor volume exceeded 500 mm^3^ (Fig. S7). Tumor growth was accelerated in the co-inoculated groups, although the difference between the three groups was not significant. Interestingly, serum IL-6 correlated more strongly with the amount of αSMA in the tumor than with tumor weight (Fig. 5E).

Two cancer groups were compared to investigate the effect of MR16-1 treatment in the cancer model: no treatment (Colon26) and cancer cells treated with MR16-1 (Colon26 + MR16-1). MR16-1 showed neither tumor suppression nor immune activation in the cancer cells alone model (Fig. S8).

### IL-6 regulates tumor immunosuppression via HIF1α activation

**Fig. 6:**
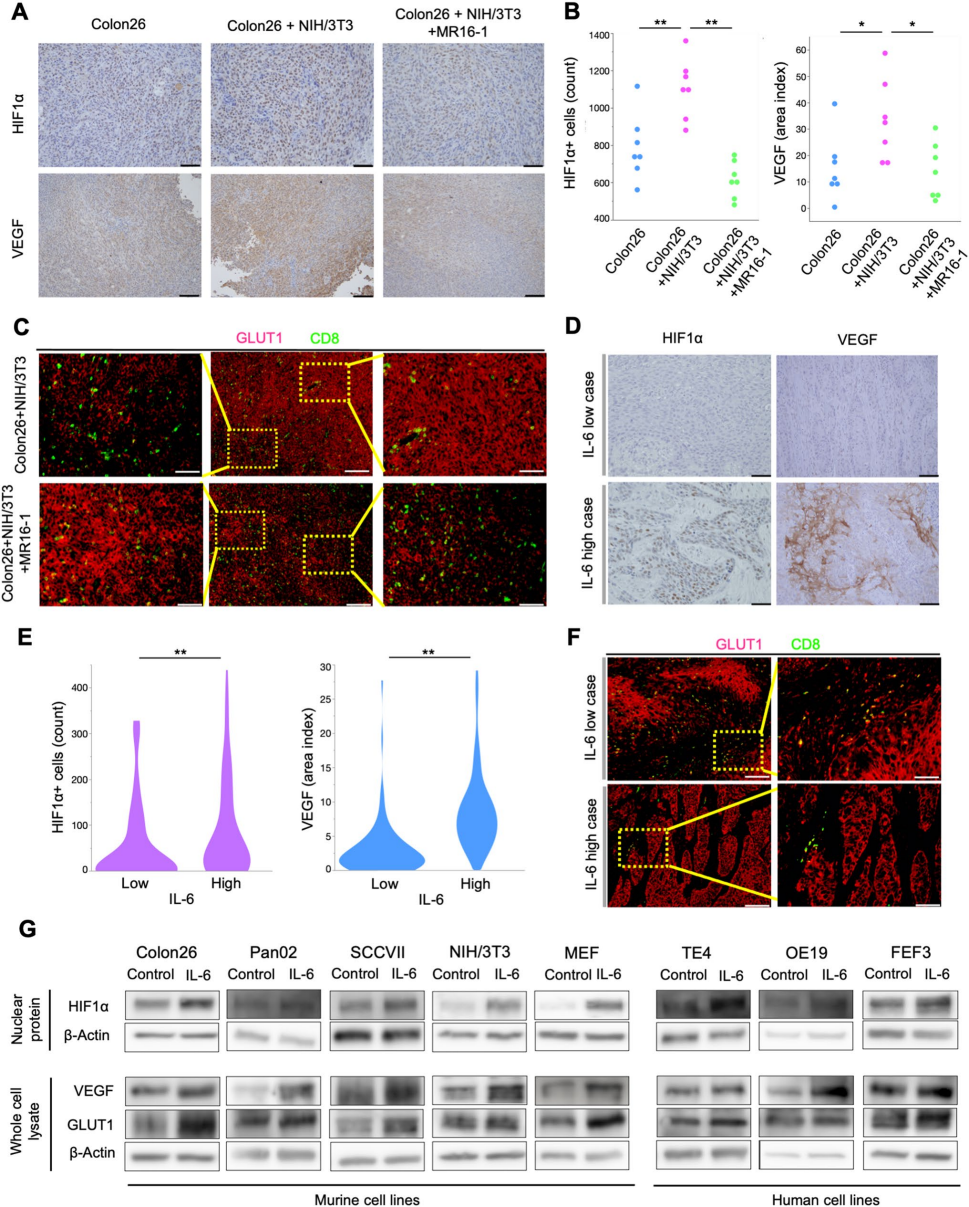
IL-6 regulates tumor immunosuppression via hypoxia/pseudohypoxia-mediated HIF1α activation. **A** IHC images for HIF1α and VEGF in tumor tissues. **B** Average number of HIF1α^+^ cells at 400× magnification and the area index of VEGF at 200× magnification. Scale bars: 100 µm (200 ×); 50 µm (400 ×). ^*^P < 0.05; ^**^P < 0.01, Tukey’s test with ANOVA. **C** IF images of GLUT-1 and CD8-expressing lymphocytes in control and MR16-1 treatment groups at 100 × and 200× magnification. Scale bars: 200 µm (100 ×), 100 µm (200 ×). **D** IHC staining for HIF1α and VEGF in human esophageal cancer tissue*s*. **E** Average number of HIF1α^+^ cells at 400× magnification and the area index of VEGF at 200× magnification. Scale bars: 100 µm (200 ×), 50 µm (400 ×). ^**^P < 0.01, Student’s *t* test. **F** IF images for GLUT-1 and CD8-expressing lymphocytes in a high and low IL-6 case at 100 × and 200× magnification. Scale bars: 200 µm (100 ×); 100 µm (200 ×). **G** Western blot analysis for HIF1α, VEGF, GLUT-1, and β-actin

To evaluate the relationship between IL-6 and HIF1α activity under hypoxic TME, HIF1α, VEGF, and glucose transporter-1 (GLUT-1), a hypoxia marker, were evaluated by IHC in the in vivo and clinical specimens. In vivo expression of HIF1α and VEGF increased in the high IL-6 state of CAFs present and decreased with MR16-1 treatment (Figs. 6A, B and S9). Although GLUT-1 expression was heterogeneous within the tumor tissue samples, it was downregulated in the MR16-1 group compared to the control. In both groups, there were significantly fewer CD8^+^ TILs at the sites of high GLUT-1 expression and more CD8^+^ TILs at the sites of low expression (Fig. 6C, Fig. S11A, B). HIF1α and VEGF expression in clinical specimens were elevated in patients with high IL-6 levels (Fig. 6D, E). The heterogeneity of GLUT-1 expression within the tumors was similar to the in vivo specimens. Patients with high IL-6 expression also showed high GLUT-1 expression and low CD8^+^ TILs, while the opposite trend was observed in patients with low IL-6 expression (Figs. 6F, S11C, D).

Next, we used western blotting to evaluate whether IL-6 activated HIF1α signaling by hypoxia-independent mechanisms. This analysis showed that IL-6 administration increased the expression of HIF1α, VEGF, and GLUT-1 in both murine and human cell lines under normoxic conditions (Figs. 6G, S12). CAFs were the major regulators of IL-6 in TME and secreted much higher levels of IL-6 than cancer cells, TAMs, or normal fibroblasts. Furthermore, IL-6 secretion was increased under hypoxia compared to normoxic conditions (Fig. S10).

## Discussion

We demonstrated that CAFs induced TILs and TAMs within the TME, into an immunosuppressed state via IL-6 to promote tumor growth and explored a mechanism of IL-6-mediated immunosuppression by CAFs. Furthermore, we showed that systemic administration of MR16-1 alleviated CAF-induced immunosuppression and suppressed tumor growth in vivo, suggesting that an anti-IL-6 receptor antibody could be used for cancer treatment. Regarding the induction of IL-6-mediated immunosuppression by CAFs, we found support for the “metabolic competition” hypothesis between T-cells and tumor cells, which results in T-cell dysfunction and immunosuppression due to the increased glucose metabolism of tumor cells caused by hypoxia-related signals, which were improved by MR16-1 treatment (Fig. S13).

Drug repositioning refers to the use of known drugs for the treatment of diseases other than those for which they were initially designed [31, 32]. TCZ was recently repurposed to treat coronavirus disease 2019 (COVID-19) caused by the severe acute respiratory syndrome coronavirus 2 [33]. Therefore, we investigated the application of TCZ in cancer treatment. In this study, we found that the primary source of IL-6 was CAFs. Additionally, we previously reported that cancer stimulus activated normal fibroblasts into CAFs and also triggered IL-6 secretion from CAFs [15]. Furthermore, CAFs created an IL-6-mediated positive feedback loop. IL-6 increased the differentiation of CAFs and TAMs, which further increased the secretion of IL-6. Meanwhile, MR16-1 treatment suppressed tumor growth by activating tumor immunity and inhibiting the growth of CAFs. However, no therapeutic effect was observed with CAF-poor models, indicating the limitations of IL-6 blockade therapy. This result may be one of the reasons that TCZ showed no significant benefit for a novel cancer therapy [22, 34, 35], suggesting that the anti-IL-6 receptor antibody treatment could be specifically effective in treating tumors with high CAF abundance, and TCZ repositioning is expected to improve the survival of patients who develop refractory cancers.

Although no effective biomarkers for estimating CAF abundance were previously described, we identified blood IL-6 levels as a potential candidate for estimating CAF abundance in the TMEs. The relationship between blood IL-6 levels and survival has been reported in various cancers [36–38], but few studies have examined this relationship histologically. In vivo*,* we demonstrated that serum IL-6 concentration in mice showed a stronger correlation with αSMA positive IHC than with tumor weight, indicating that the amount of IL-6-producing CAFs in the TME influences tumor development. Although this trend was observed in other cell types, the difference was not significant, suggesting that the amount of IL-6 produced by CAFs varies from cell to cell. Since our results highlight blood IL-6 concentration as a potential biomarker of CAFs, as well as a predictor of anti-IL-6 receptor antibody efficacy, further analyses using clinical specimens are warranted.

IL-6 suppresses immune functions in TME by increasing competition between tumors and T-cells for glucose in hypoxic TME. CD8^+^ TILs demonstrate cytotoxicity toward tumor cells, while FoxP3^+^ TILs and CD163^+^ TAMs suppress antitumor immunity, contributing to tumor progression [39, 40]. The mechanism by which IL-6 suppresses T-cells remains unclear. Although IL-6 suppresses the function of Tregs [41], our results showed the opposite. To investigate this contradiction, we focused on hypoxic TME. Tumor hypoxia forms in advanced cancers with actively proliferating cells, and CTL numbers are reduced due to glucose deficiency. Meanwhile, Treg and M2 macrophage numbers increase by using oxidized lipids as a fuel source under hypoxic conditions, leading to an immunosuppressive state [42–44]. Our results demonstrate that CAFs are the major regulators of IL-6 in TME and IL-6 increased cell proliferation, while IL-6 production by CAFs was enhanced under hypoxia, suggesting that IL-6 and hypoxia exert mutually positive feedback.

Although most solid tumors have hypoxic regions, not all regions or tumors are hypoxic, and it is difficult to attribute cancer pathogenesis to hypoxia. Evidence has revealed various hypoxia-independent mechanisms for HIF1α signaling activation, which are termed “pseudohypoxia” [45]. We observed that HIF1α expression was upregulated by the addition of IL-6 in normoxic conditions, while VEGF and GLUT-1 were continuously upregulated. IL-6 is known to increase the transcriptional activity of HIF1α via signal transducer and activator of transcription3 (STAT3) signaling under hypoxia, and furthermore, HIF1α upregulate VEGF expression via STAT3 pathway and activate GLUT-1 via phosphatidylinositol-3 kinase (PI3K) pathway [46–48]. We revealed that IL-6 regulated HIF1α activation through a hypoxia-independent mechanism [49]. On the other hand, HIF1α, VEGF, and GLUT-1 expression correlated with IL-6 expression in clinical samples and decreased following MR16-1 treatment in vivo, which may reflect hypoxia-mediated HIF1α activation. In tumor tissues, HIF1α shifts glucose metabolism from oxidative phosphorylation to anaerobic processes (the Warburg effect) [50–52]. GLUT-1 upregulation accompanying accelerated glucose metabolism in the tumor is associated with low infiltration of effector T-cells [53]. Meanwhile, HIF1α and VEGF inhibit the development and activation of CTLs while increasing the number and immunosuppressive functions of Tregs and TAMs [54, 55]. IL-6 might induce tumor immunosuppression by decreasing effector T-cells by enhancing cancer glucose uptake and by increasing regulatory cells through HIF1α and VEGF function via hypoxia-pseudohypoxia-mediated HIF1α activation. Therefore, CAFs would mediate tumor immunosuppression by regulating hypoxia-pseudohypoxia-mediated HIF1α activation via IL-6. Furthermore, IL-6 secretion was increased under hypoxia [56–58] (supplementary Fig. S10D), and HIF1α itself is also known to upregulate IL-6 expression [59, 60]. Thus, there would be a positive feedback loop between IL-6 signaling and HIF-1α expression in the TME.

Our study revealed some interesting results, but also has limitations. First, it is known that IL-6 has two signaling pathways, classical signaling and trans-signaling, and we evaluated the effects of trans-signaling of cancers and fibroblasts on tumor immunity by simultaneous administration of IL-6 and IL-6R [61–63] In this study, the effect of IL-6 via classical signaling on cells originally expressing membrane IL-6R, such as B cells or myeloid cells, was not evaluated. Further additional effects may be observed by assessing the tumor immunity generated by these cells. Second, we demonstrated that HIF1α was elevated in both allograft models and clinical specimens with high IL-6 expression, and anti-IL-6R decreased HIF1α expression in vivo models, suggesting improvement of hypoxia. However, the mechanism of the direct relationship between anti-IL-6 receptor antibody and local hypoxia is still unclear, therefore further investigation is required. Finally, the TCZ analog (MR16-1) was the used to evaluate the effects of the anti-IL-6 receptor antibody. Future trials are needed to evaluate the exact effects of TCZ on cancer treatment.

In conclusion, we demonstrated that CAFs are the major regulators of IL-6 in the TME, and blood IL-6 concentration could be a potential biomarker of CAFs, while systemic administration of an anti-IL-6 receptor antibody overcomes CAF-induced immunosuppression and halts tumor progress. Furthermore, we described the mechanism by which IL-6 mediates tumor immunosuppression by focusing on metabolic competition between T-cells and tumor cells via hypoxia-pseudohypoxia-mediated HIF1α activation. Hence, the anti-IL-6 receptor antibody may be applied for treating tumors with high CAF abundance, overcoming tumor immunosuppression and improving the survival of patients with various cancers.

### Supplementary Information

Below is the link to the electronic supplementary material.

Supplementary file1 (DOCX 57073 KB)

## Abbreviations

BMDM: Bone marrow-derived monocytes
CAF: Cancer-associated fibroblast
CI: Confidence interval
COVID-19: Coronavirus Disease 2019
CTL: Cytotoxic T-cells
DFS: Disease-free survival
EC: Esophageal cancer
EMR: Endoscopic mucosal resection
ESD: Endoscopic submucosal dissection
FoxP3: Forkhead box protein 3
GLUT: Glucose transporter
HIF1α: Hypoxia-inducible factor 1α
HR: Hazard ratio
Iba1: Ionized calcium-binding adaptor protein 1
IF: Immunofluorescence
IHC: Immunohistochemistry
IL: Interleukin
IL-6Rα: Interleukin-6 receptor alpha
JCRB: Japanese Collection of Research Bioresources
MDSC: Myeloid-derived suppressor cell
M-CSF: Mouse macrophage colony-stimulating factor
OS: Overall survival
PI3K: Phosphatidylinositol-3 kinase
PMA: Phorbol-12-myristate-13-acetate
RT: Room temperature
SE: Standard error
SMA: Smooth muscle actin
STAT: Signal transducer and activator of transcription
TAM: Tumor-associated macrophage
TCZ: Tocilizumab
TIL: Tumor-infiltrating lymphocyte
TME: Tumor microenvironment
Treg: Regulatory T-cell
TTDR: Tumor and Tissue Dissociation Reagent
UICC: Union for international cancer control
VEGF: Vascular endothelial growth factor
XTT: Sodium 2,3,-bis (2-methoxy-4-nitro-5-sulfophenyl)-5-[(phenylamino)-carbonyl]-2H-tetrazolium
