# Supplementary material for: Overcoming cancer-associated fibroblast-induced immunosuppression by anti-interleukin-6 receptor antibody
Source: Cancer Immunol Immunother. 2023 Feb 10;72(7):2029–44. doi: 10.1007/s00262-023-03378-7 (PMC9916502; doi:10.1007/s00262-023-03378-7)
Supplement: Supplementary file 1 — Supplementary file1 (DOCX 57073 KB) [file 262_2023_3378_MOESM1_ESM.docx]

**
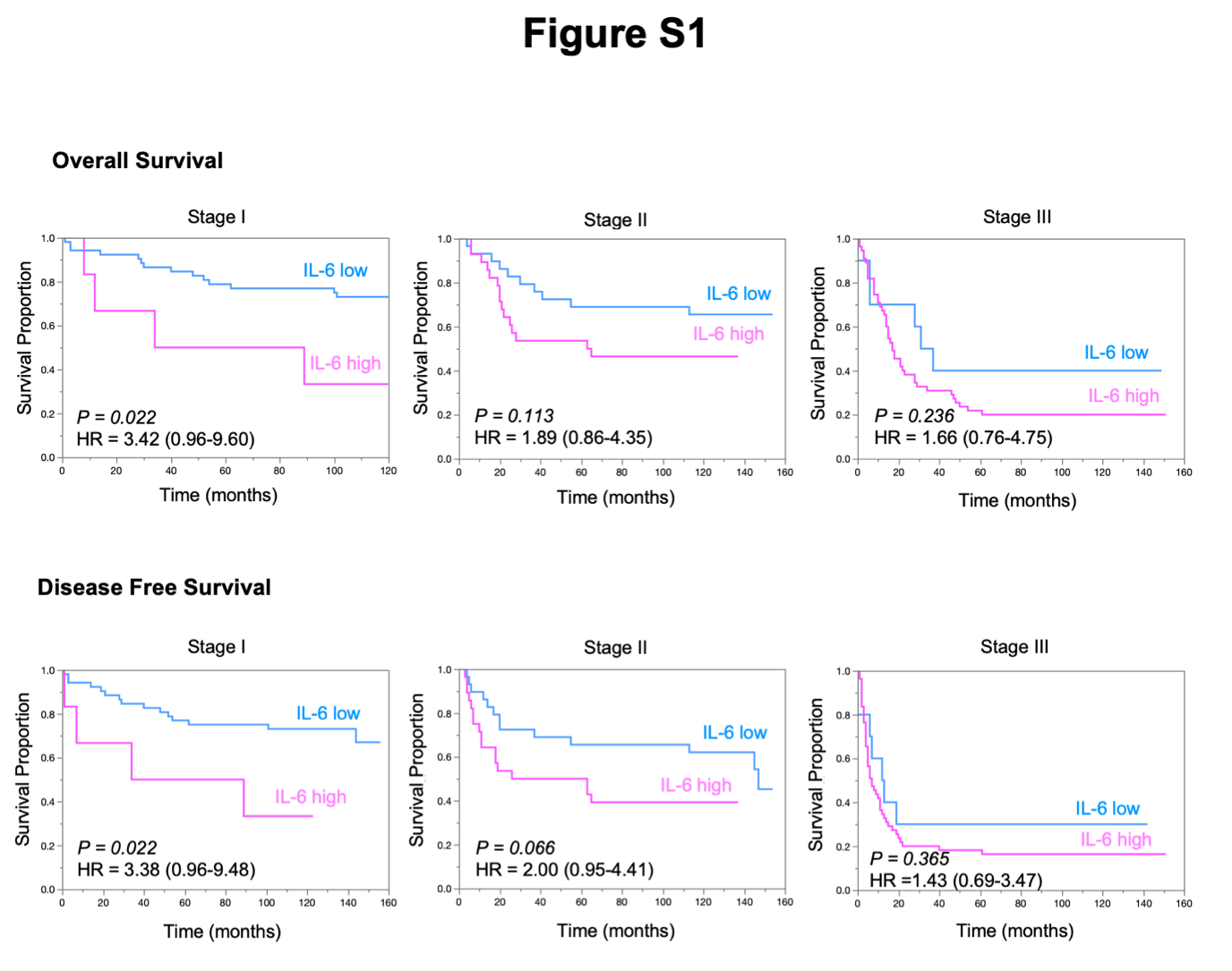
**

**Figure S1. Subgroup analysis of IL-6 expression according to UICC Stage.**

Survival curve according to IL-6 expression (low or high group). The low IL-6 group showed a significantly better prognosis at all stages (Cox regression hazard model, 95% confidence intervals, and log-rank test).


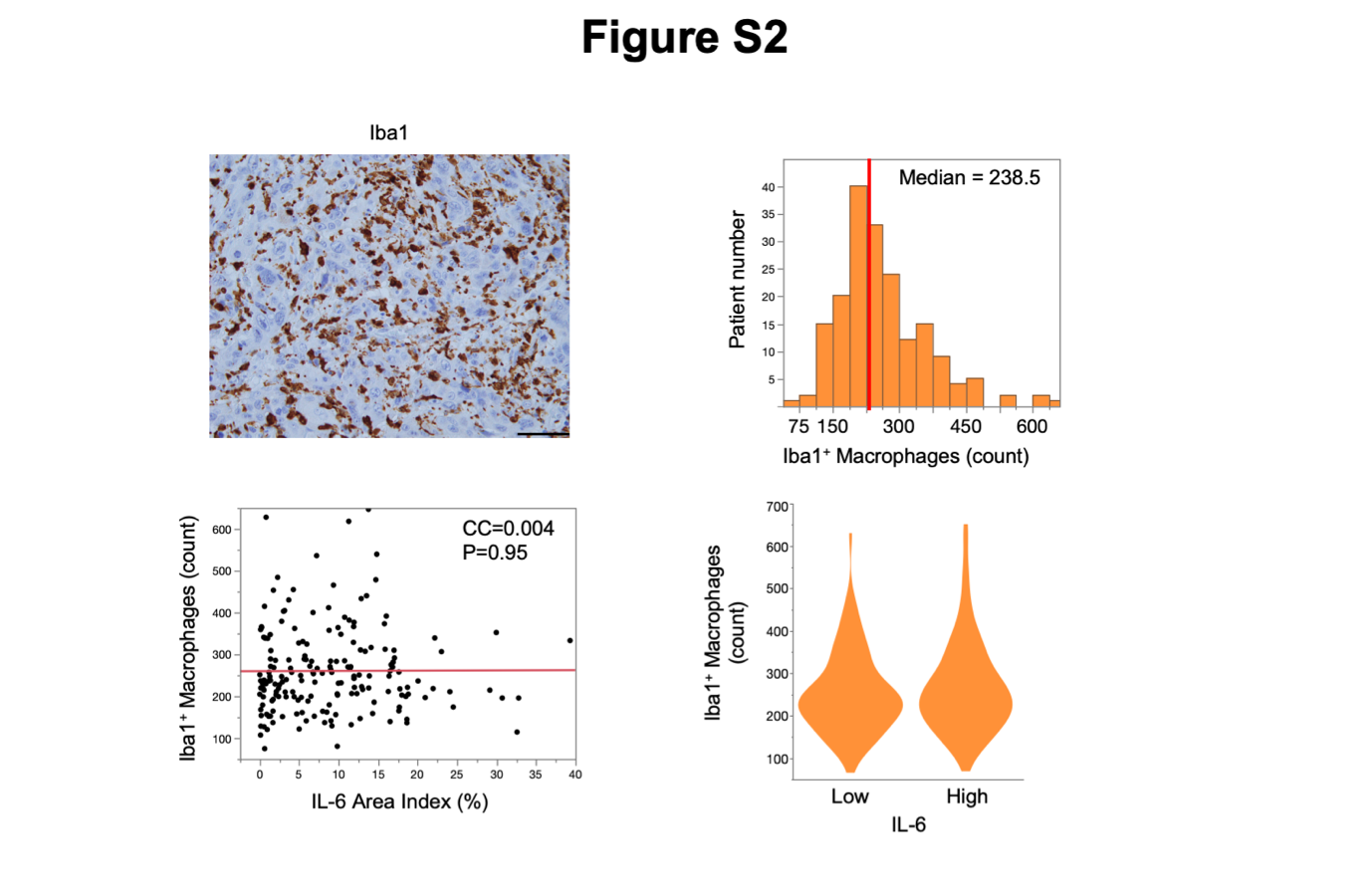


**Figure S2. Correlation of IL-6 expression and TAMs distribution in esophageal cancer tissues.**

The average number of Iba1^+^ TAMs at high magnification (400×) was recorded using ImageJ. Scale bars: 50 µm (400×). The correlation between IL-6 and Iba1^+^ TAMs is shown by scatter plot. The violin plots show comparisons based on high or low IL-6 area index.

**
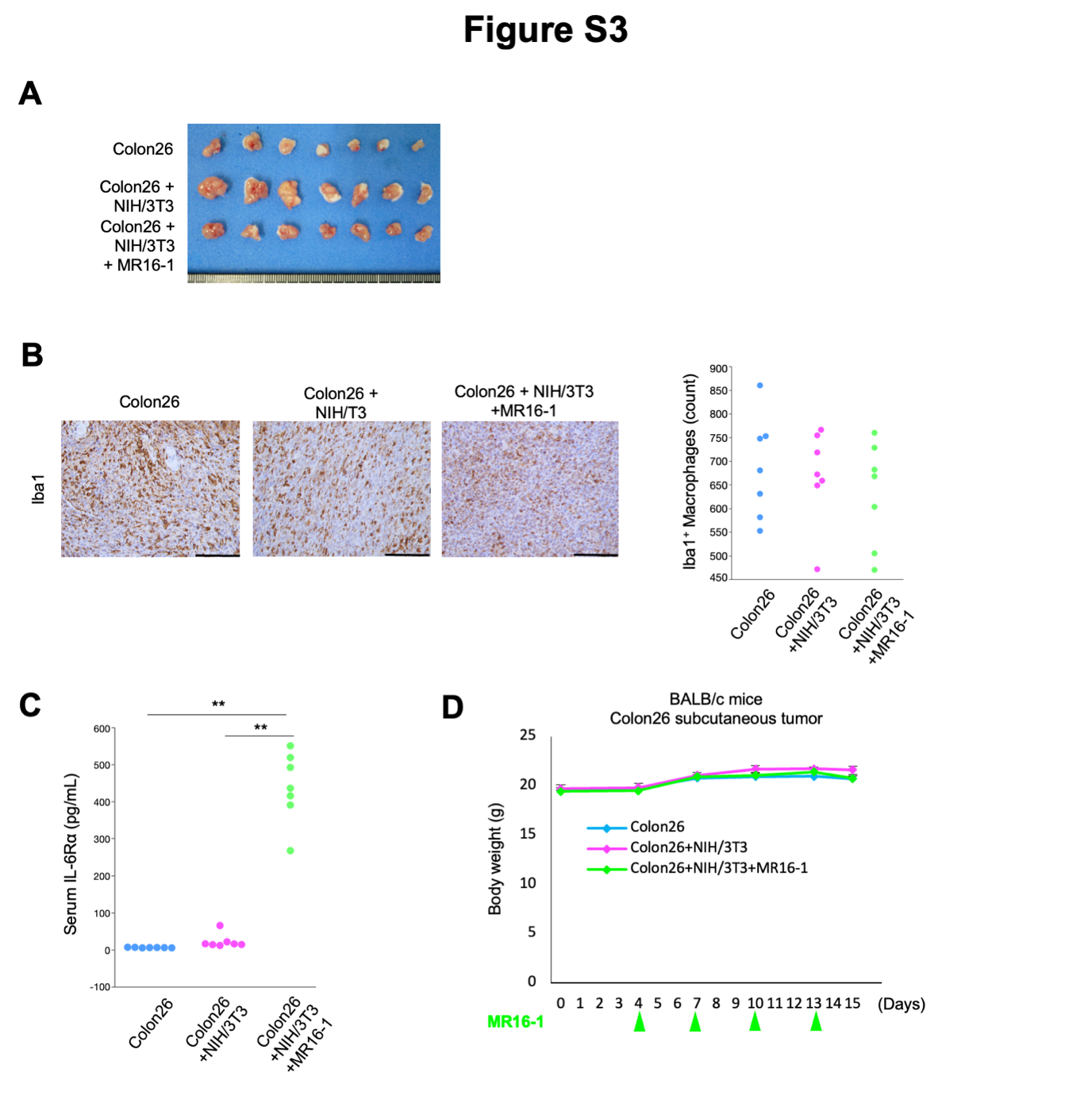
**

**Figure S3. MR16-1 treatment for Colon26 + NIH/3T3 subcutaneous tumors in BALB/c mice.**

(A) Macroscopic findings of harvested tumors; n = 7 mice/group. (B) Representative figures of immunohistochemical staining for Iba1 in tumor tissues. The average number of Iba1^+^ TAMs at 400× magnification was recorded using ImageJ. Scale bars: 50 µm. (C) Quantification of serum IL-6Rα concentration by ELISA. ^**^*P* < 0.01, Tukey’s test with ANOVA. (D) The mean body weights for each group.


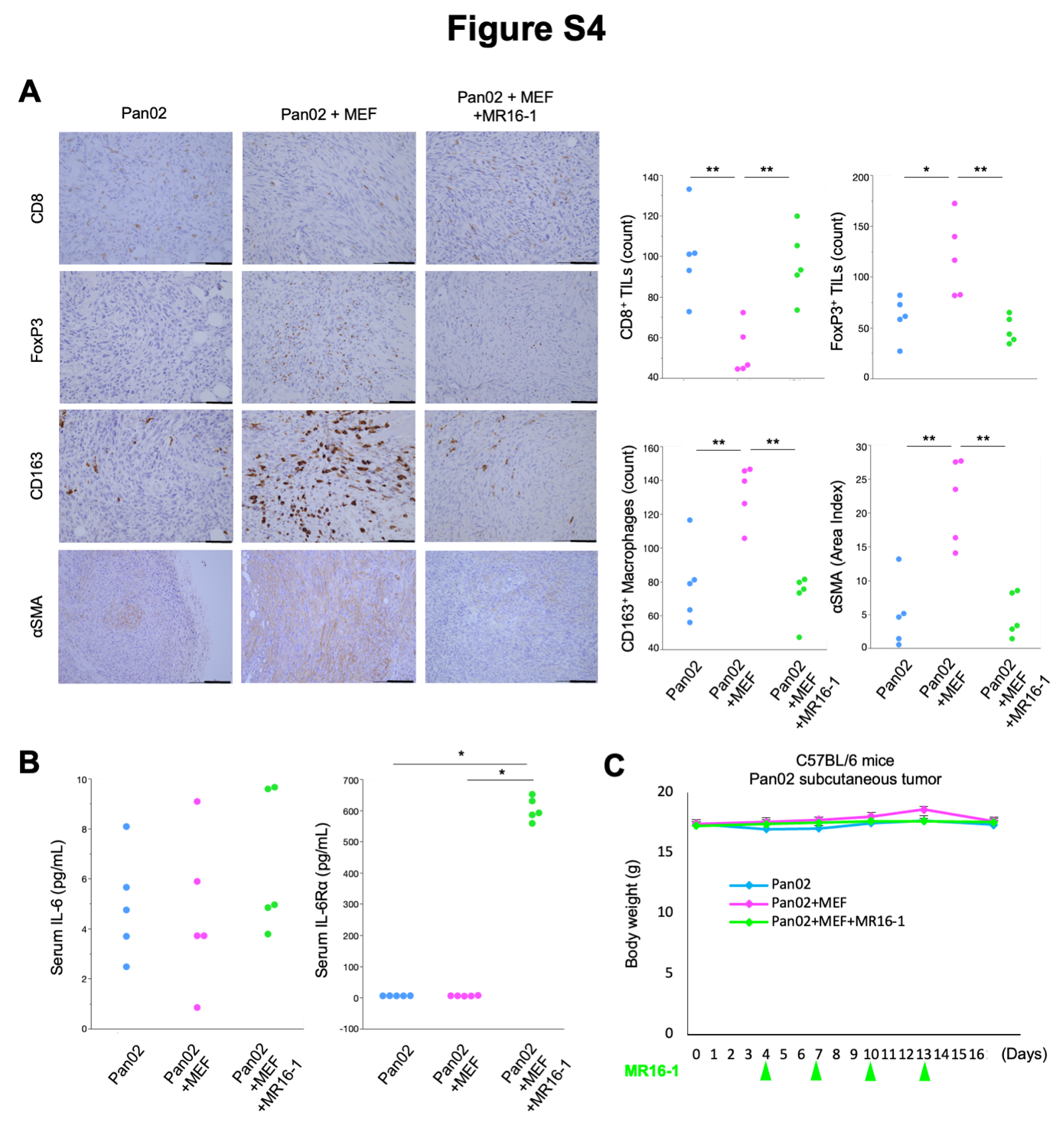


**Figure S4. MR16-1 treatment for Pan02 + MEF subcutaneous tumors in C57BL/6 mice.** (A) Representative figures of immunohistochemical staining for CD8, FoxP3, CD163, and αSMA in tumor tissues. The average number of CD8^+^ or FoxP3^+^ TILs and CD163^+^ TAMs at 400× magnification and the area index of αSMA at 200× magnification were recorded using ImageJ. Scale bars: 100 µm (200×), 50 µm (400×). ^*^*P* < 0.05; ^**^*P* < 0.01, Tukey’s test with ANOVA. (B) Quantification of serum IL-6 and IL-6Rα concentration by ELISA. ^*^*P* < 0.05. (C) Mean body weights for each group.

**
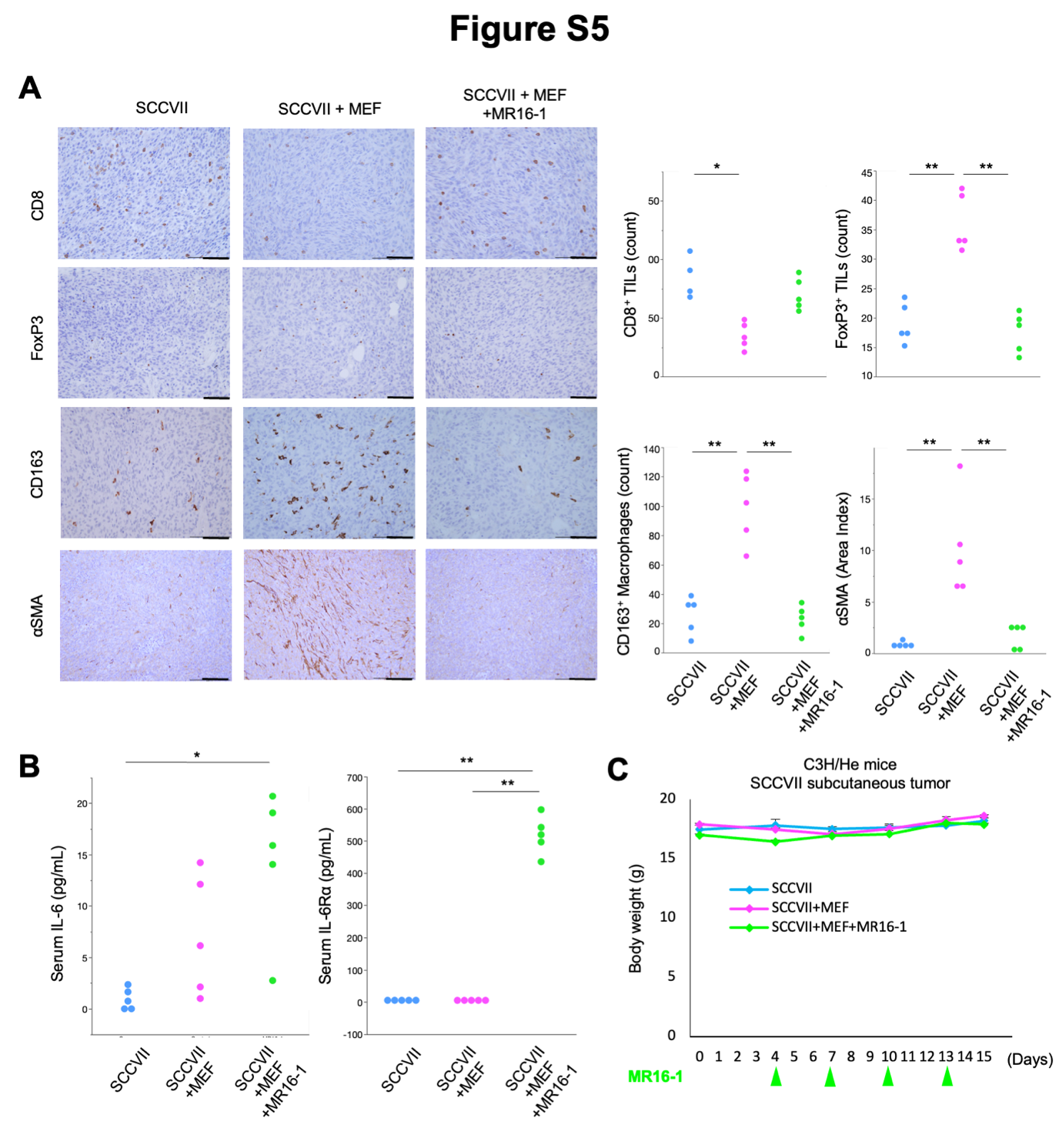
Figure S5. MR16-1 treatment for SCCVII + MEF subcutaneous tumors in C3H/He mice.** (A) Representative immunohistochemical staining images for CD8, FoxP3, CD163, and αSMA in tumor tissues. The average number of CD8^+^ or FoxP3^+^ TILs and CD163^+^ TAMs at 400× magnification and the area index of αSMA at 200× magnification were recorded using ImageJ. Scale bars: 100 µm (200×), 50 µm (400×). ^*^*P* < 0.05; ^**^*P* < 0.01, Tukey’s test with ANOVA. (B) Quantification of serum IL-6 and IL-6Rα concentration by ELISA. ^*^*P* < 0.05, Tukey’s test with ANOVA. (C) Mean body weights of each group.

**
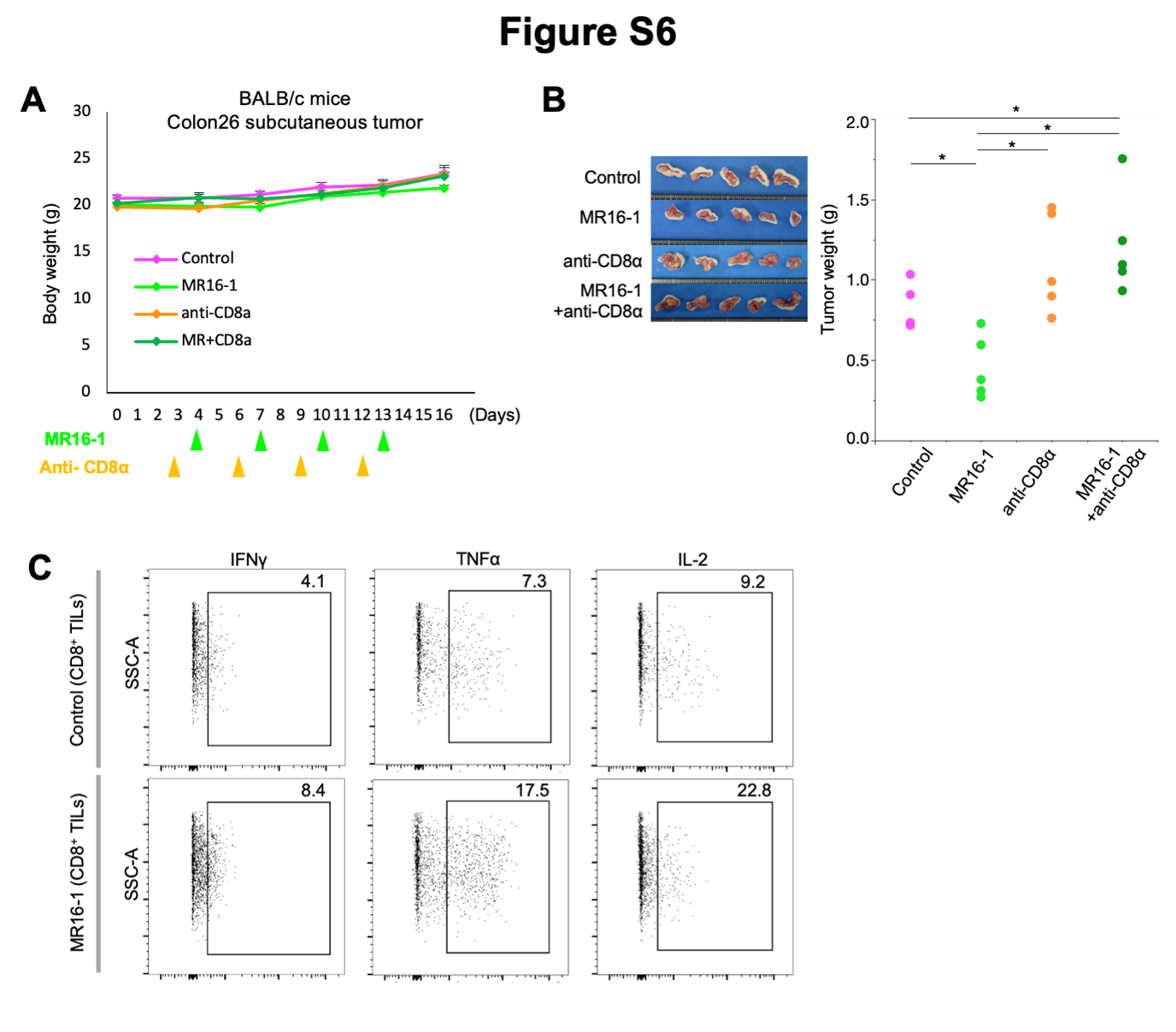
**

**Figure S6 Treatment with MR16-1 and anti-CD8**α **antibody for Colon26 + NIH/3T3 subcutaneous tumors in BALB/c mice.**

(A) Mean body weights for each group. (B) Macroscopic findings and weights of harvested tumors; n = 5 mice/group; mean ± SE. ^*^*P* < 0.05, Tukey’s test with ANOVA. (C) Representative figures of flow-cytometric analysis: cells in the area surrounded by the black border are positive cells.


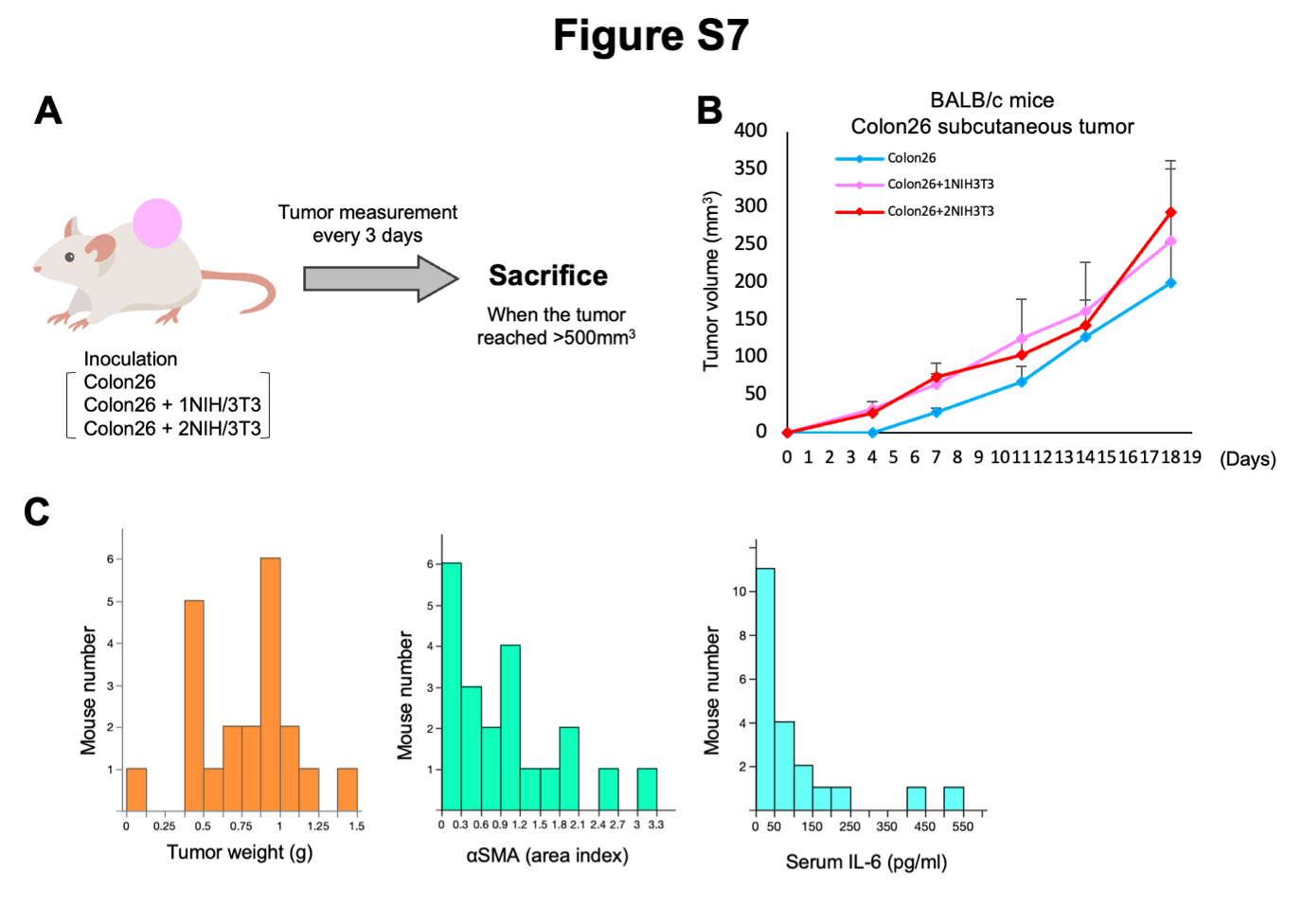


**Figure S7. Protocol to assess the relationship between CAFs and serum IL-6 in Colon26 + NIH/3T3 subcutaneous tumors.**

(A) Study protocol. In brief, three groups with varying amounts of co-inoculated fibroblasts of Colon26 subcutaneous tumors were harvested after the tumor volume exceeded 500 mm^3^. (B) Tumor volume of the transplanted mice in each group until 18 days after inoculation. (C) Tumor weight, αSMA area index, and IL-6 area index for all mice.


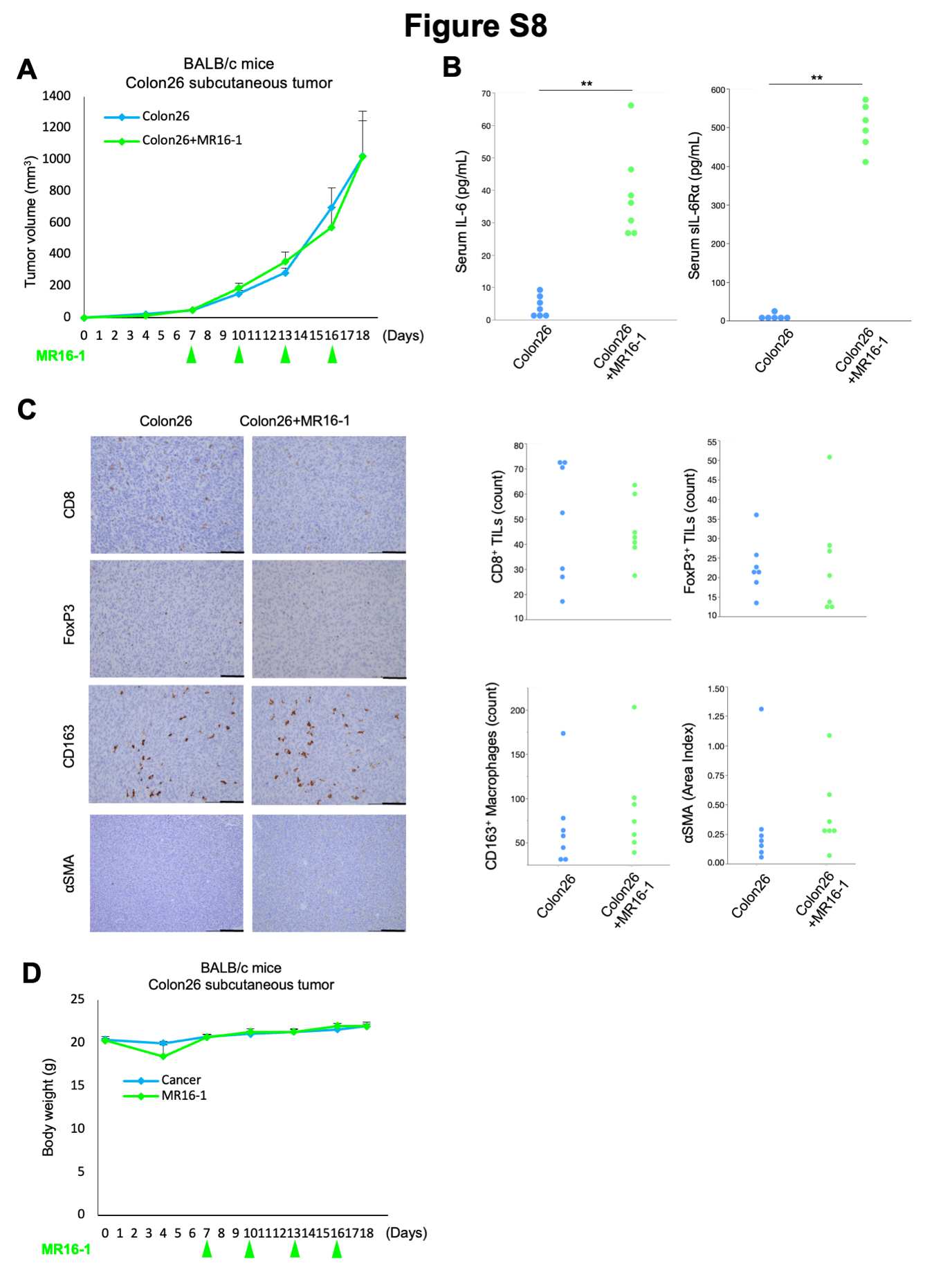


**Figure S8. MR16-1 treatment for Colon26 subcutaneous tumors in BALB/c mice.**

(A) Tumor volume in the transplanted mice, with or without MR16-1 treatment; n = 7 mice/group; mean ± SE. (B) Quantification of serum IL-6 and IL-6Rα concentration by ELISA. ^*^*P* < 0.05; ^**^*P* < 0.01, Student’s *t*-test. (C) Representative immunohistochemical staining for CD8, FoxP3, CD163, and αSMA in tumor tissues. Average number of CD8^+^ or FoxP3^+^ TILs and CD163^+^ TAMs at 400× magnification and the area index of αSMA at 200× magnification were recorded using ImageJ. Scale bars: 100 µm (200×), 50 µm (400×). (D) Mean body weights of each group.

**
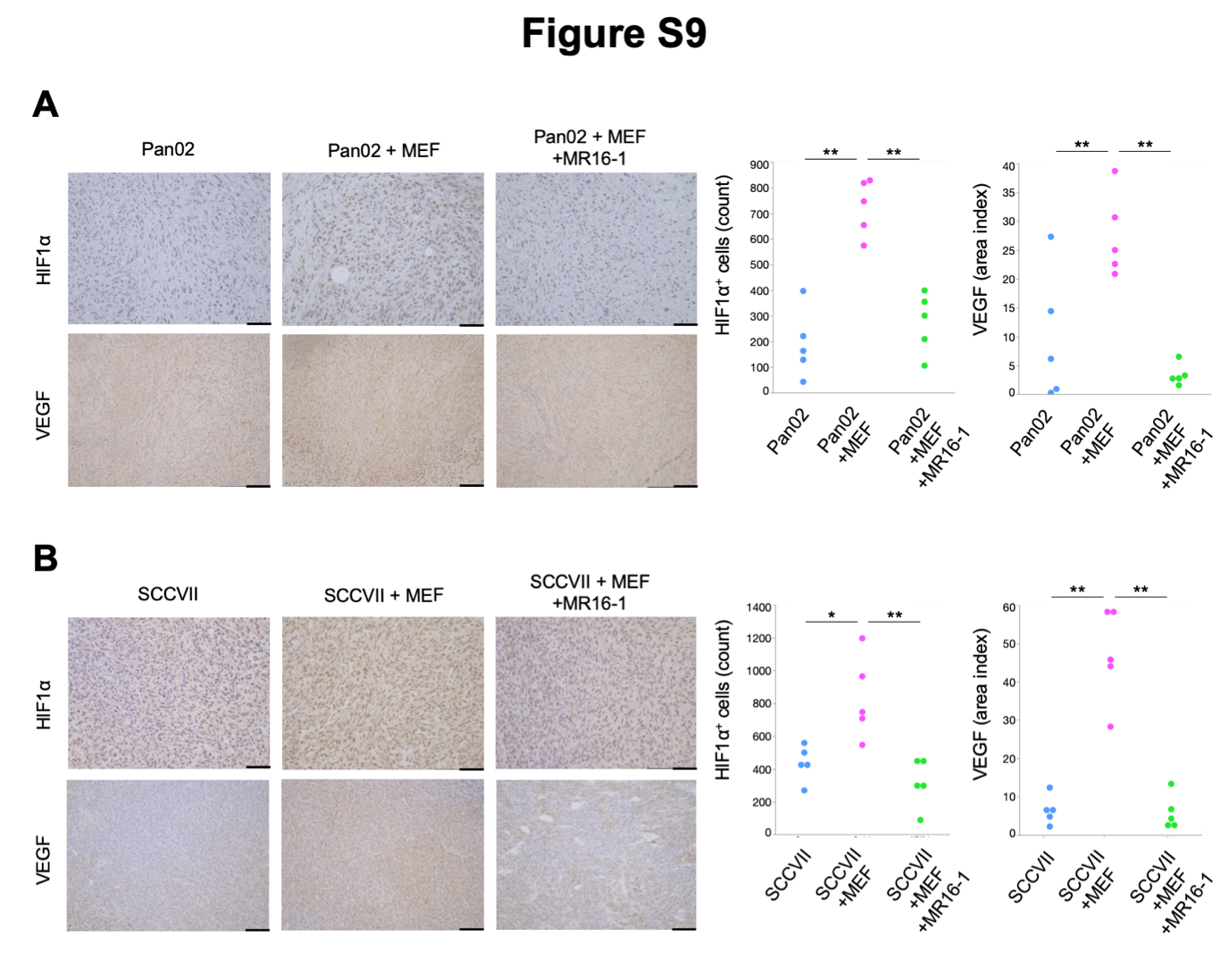
**

**Figure S9. HIF1α and VEGF expression in Pan02 and MEF subcutaneous tumor models.**

(A, B) Representative immunohistochemical staining for HIF1α and VEGF in tumor tissues. Average number of HIF1α^+^ cells at 400× magnification and the area index of VEGF at 200× magnification were recorded using ImageJ software. Scale bars: 100 µm (200×), 50 µm (400×). ^*^*P* < 0.05; ^**^*P* < 0.01, Tukey’s test with ANOVA. (A) Pan02 + MEF model (B) SCCVII + MEF model.

**
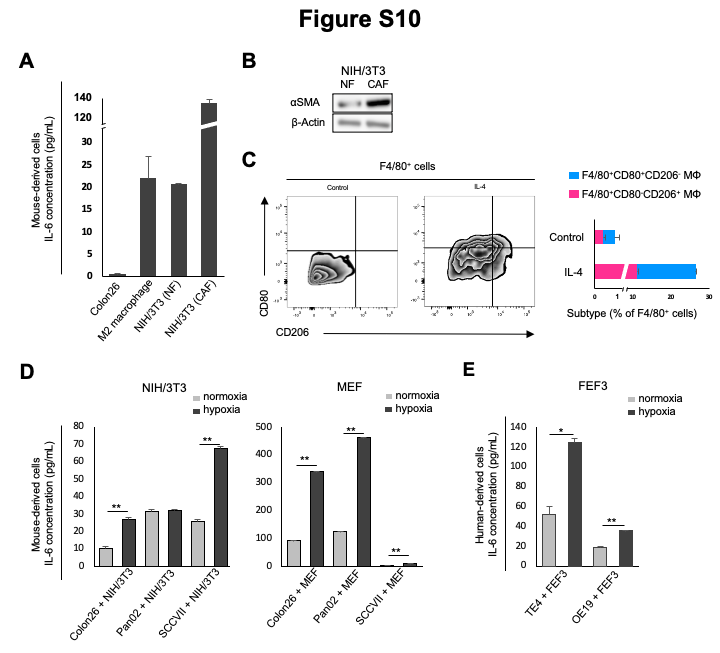
**

**Figure S10. Cancer-associated fibroblasts are the main source of IL-6 secretion, which promote under hypoxia.**

(A) Quantification of IL-6 secretion by ELISA. Cancer cells (Colon26: 0.5 × 10^6^ cells), M2 macrophages (BMDM stimulated by IL-4: 0.5 × 10^6^ cells), normal fibroblasts (NFs) (NIH/3T3: 0.5 × 10^6^ cells), cancer-associated fibroblasts (CAFs) (NIH/3T3 stimulated by conditioned medium of Colon26: 0.5 × 10^6^ cells) were seeded in 6-well plates and cultured with 10% DMEM. Culture supernatant was collected 48 h later. Data are presented as IL-6 levels per 0.1 × 10^6^ cells. (B) Western blot of αSMA expression in NFs and CAFs. (C) Flow cytometry analysis of F4/80 (M1/M2 marker), and CD80 (M1 marker) on the cell surface, and intracellular CD206 (M2 marker) expression in BMDMs, with or without IL-4 (20 ng/mL) treatment for 2 days. The bar chart shows quantification of the F4/80^+^, CD80^+^, and CD206^-^ (M1) population and F4/80^+^, CD80^-^, and CD206^+^ (M2) populations; n = 3. (D and E) Quantification of IL-6 secretion in co-culture model under normoxia and hypoxia. Cancer cells (0.1 × 10^6^ cells) and fibroblasts (0.1 × 10^6^ cells) were seeded in 6-well plates and cultured with 10% DMEM. Cells were incubated under normoxic conditions at 37 °C in a humidified atmosphere with 5% CO_2_ and 20% O_2_. Cells were incubated in a hypoxic chamber (Modular Incubator Chamber; Billups-Rothenberg) filled with a gas mixture of 1% O_2_, 5% CO_2_, and N_2_. Culture supernatant was collected 48 h later. (D) Murine cell lines. (E) Human cell lines. Data are presented as IL-6 levels per 0.1 × 10^6^ cells. ^*^*P* < 0.05; ^**^*P* < 0.01, Tukey’s test with ANOVA.


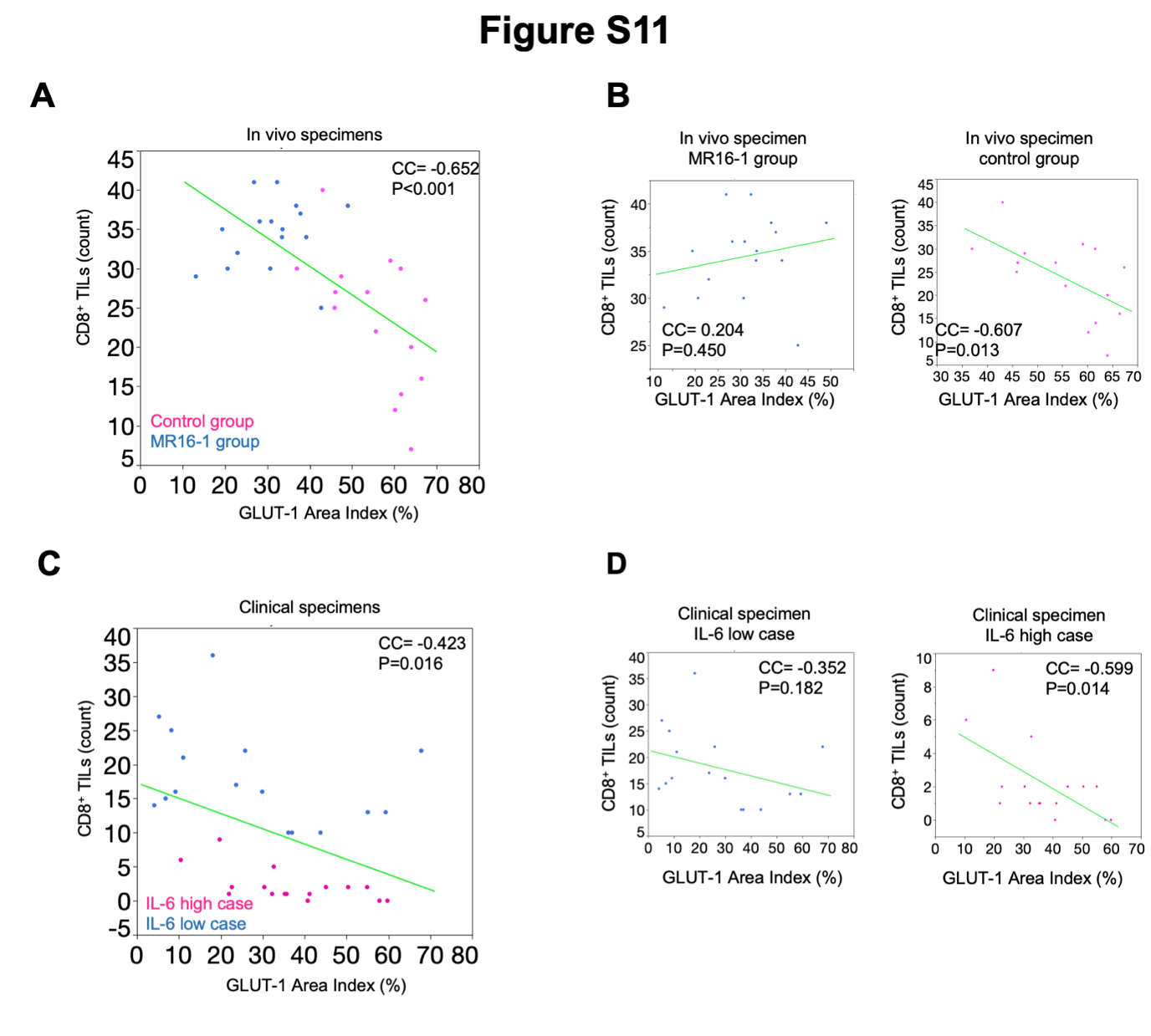


**Figure S11. The relationship between GLUT-1 expression and CD8-expressing lymphocytes in the tumor microenvironment.** The number of cells expressed CD8 and GLUT-1 area index in high magnification fields of immunofluorescence images were analyzed with Image J (NIH). (B, C) The correlation between GLUT-1 and CD8-expressing lymphocytes in contol and MR16-1 treatment groups are shown by scatter plot. (D, E) The correlation between GLUT-1 and CD8-expressing lymphocytes in human esophageal cancer tissues are shown by scatter plot.


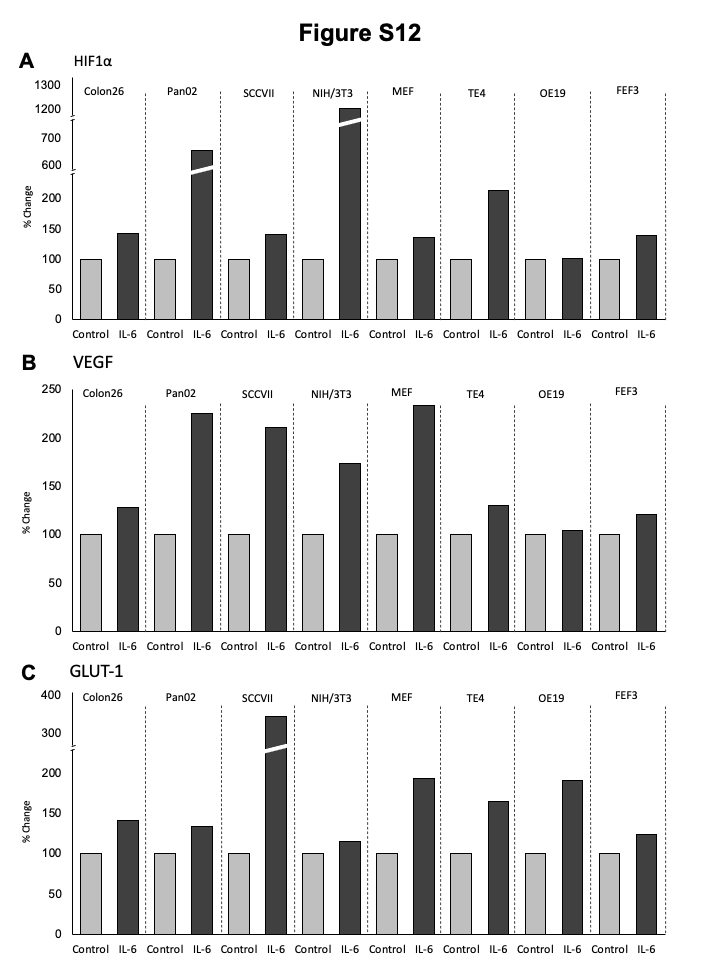


**Figure S12. Densitometry of the western blot analysis.**

Densitometry of the western blot analysis in Figure 6G was analyzed with Image J software (NIH). The expression was normalized to β-actin expression measured in the same sample as an internal control. Changes are shown in percent, compared to control. (A) HIF1α, (B) VEGF, (C) GLUT-1.

**Figure S13**

**
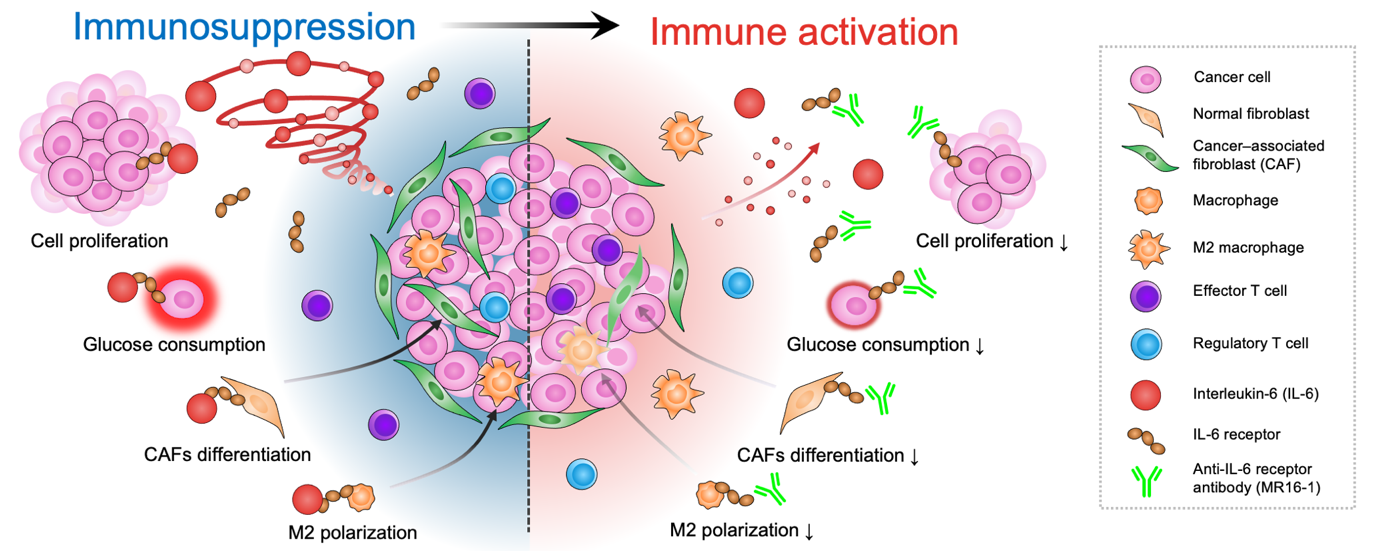
**

**Figure S13. Schematic illustration of the effect of MR16-1 in the tumor microenvironment.**

CAFs contribute to tumor progression by inducing immunosuppression via IL-6 in EC patients while MR16-1 treatment overcomes CAF-induced immunosuppression and halts tumor progress.

**Table S1. List of antibodies used in this study.**

| Antibody | Application | Dilution | Company | Catalog no. |
| --- | --- | --- | --- | --- |
| Anti-CD8 antibody | IHC (clinical specimens) | 1:100 | Dako | M7103 |
| Anti-FOXP3 antibody [236A/E7] | IHC (clinical specimens) | 1:100 | Abcam | ab20034 |
| Anti-Actin, α-Smooth Muscle antibody | IHC (clinical specimens, vivo specimens) | 1:1000 | Sigma-Aldrich | A5228 |
| Anti-alpha smooth muscle Actin antibody [1A4] (FITC) | IF (clinical specimens, vivo specimens) | 1:100 | Abcam | ab8211 |
| Anti-IL-6 antibody | IHC (clinical specimens) | 1:4000 | Abcam | ab9324 |
| Anti-Iba1 antibody [EPR16588] | IHC (clinical specimens, vivo specimens) | 1:2000 | Abcam | ab178846 |
| Anti-CD163 antibody [EPR19518] | IHC (clinical specimens, vivo specimens) | 1:500 | Abcam | ab182422 |
| Purified Mouse Anti-Human HIF-1α | IHC (clinical specimens) | 1:50 | BD Biosciences | 610959 |
| Anti-VEGFA antibody [EP1176Y] - C-terminal | IHC (clinical specimens) | 1:100 | Abcam | ab52917 |
| CD8a Monoclonal Antibody (4SM15) | IHC (vivo specimens) | 1:100 | eBioscience | 14-0808-82 |
| FOXP3 Monoclonal Antibody (FJK-16s) | IHC (vivo specimens) | 1:100 | eBioscience | 14-5773-82 |
| HIF1α | IHC (vivo specimens) | 1:1000 | Novus | NB100-296 |
| VEGF | IHC (vivo specimens) | 1:1000 | Abcam | ab232858 |
| Alexa Fluor® 488 F(ab')2 fragment of goat anti-mouse IgG (H+L) | IF (clinical specimens) secondary antibody | 1:500 | Thermo Fisher Scientific | A11017 |
| Alexa Fluor® 568 F(ab')2 fragment of goat anti-mouse IgG (H+L) | IF (clinical specimens) secondary antibody | 1:500 | Thermo Fisher Scientific | A11019 |
| Alexa Fluor® 647 F(ab')2 fragment of goat anti-mouse IgG (H+L) | IF (clinical specimens) secondary antibody | 1:500 | Thermo Fisher Scientific | A21237 |
| Alexa Fluor® 647 F(ab')2 fragment of goat anti-rabbit IgG (H+L) | IF (clinical specimens) secondary antibody | 1:500 | Thermo Fisher Scientific | A21245 |
| Goat anti-Rat IgG (H+L) Cross-Adsorbed Secondary Antibody, Alexa Fluor 488 | IF (*vivo* specimens) secondary antibody | 1:500 | Thermo Fisher Scientific | A11006 |
| Goat anti-Rat IgG (H+L) Cross-Adsorbed Secondary Antibody, Alexa Fluor 647 | IF (*vivo* specimens) secondary antibody | 1:500 | Thermo Fisher Scientific | A21247 |
| α-Smooth Muscle Actin (D4K9N) XP® Rabbit mAb | Western blotting | 1:1000 | Cell Signaling Technology | 19245 |
| HIF-1α (D2U3T) Rabbit mAb | Western blotting | 1:1000 | Cell Signaling Technology | 14179 |
| Anti VEGF antibody | Western blotting | 1:1000 | Proteintech | 19003-1-AP |
| Anti-Glucose Transporter GLUT1 antibody [EPR3915] | Western blotting | 1:1000 | Abcam | ab115730 |
| βactin | Western blotting | 1:1000 | Sigma-Aldrich | A5441 |
| Zombie Aqua™ Fixable Viability Kit | FACS | 1:100 | BioLegend | 423102 |
| FITC anti-mouse CD8a Antibody | FACS | 1:100 | BioLegend | 100706 |
| Brilliant Violet 421™ anti-mouse TNF-α Antibody | FACS | 1:100 | BioLegend | 506327 |
| APC anti-mouse IL-2 Antibody | FACS | 1:100 | BioLegend | 503809 |
| APC/Cyanine7 anti-mouse IFN-γ Antibody | FACS | 1:100 | BioLegend | 505849 |
| Alexa Fluor® 647 anti-mouse CD80 Antibody | FACS | 1:100 | BioLegend | 104718 |
| PE/Cyanine7 anti-mouse F4/80 Antibody | FACS | 1:100 | BioLegend | 123114 |
| PE anti-mouse CD206 (MMR) Antibody | FACS | 1:100 | BioLegend | 141706 |
| Isotype control for Rat IgG1 | *in vivo* experiment |  | BioxCell | BE0088 |
| InVivoMab anti-mouse CD8α | *in vivo* experiment |  | BioxCell | BE0061 |

**Table S2. Clinicopathological characteristics of esophageal cancer patients, according to Interleukin-6 (IL-6) status**

|  |  | **IL-6** | |  |
| --- | --- | --- | --- | --- |
| **Variables** | **Total** | **Low (n = 92)** | **High (n = 93)** | ***P* value** |
| Age (years) |  |  |  | 0.575§ |
| Median (IQR) | 66 (61–72) | 66 (61–72) | 66 (61–71) |  |
| Sex |  |  |  | 0.073† |
| Male | 163 (87.6%) | 77 (83.7%) | 86 (92.5%) |  |
| Female | 22 (12.3%) | 15 (16.3%) | 7 (7.5%) |  |
| Histological type |  |  |  | 0.795† |
| SCC | 169 (27.3%) | 85 (92.4%) | 84 (90.3%) |  |
| Adenocarcinoma | 16 (72.7%) | 7 (7.6%) | 9 (9.7%) |  |
| Neoadjuvant therapy |  |  |  | < 0.001†* |
| None | 141 (41.3%) | 81 88.0%) | 60 (64.5%) |  |
| Chemotherapy | 31 (13.2%) | 6 (6.5%) | 25 (26.9%) |  |
| Chemoradiotherapy | 13 (35.5%) | 5 (5.4%) | 8 (8.6%) |  |
| Pathological T stage |  |  |  | < 0.001†* |
| T1 | 77 (41.3%) | 68 (73.9%) | 9 (9.7%) |  |
| T2 | 23 (13.2%) | 10 (10.9%) | 13 (14.0%) |  |
| T3 | 79 (35.5%) | 13 (14.1%) | 66 (71.0%) |  |
| T4 | 6 (9.9%) | 1 (1.1%) | 5 (5.4%) |  |
| Pathological N stage |  |  |  | < 0.001†* |
| N0 | 84 (53.7%) | 62 (67.4%) | 22 (23.7%) |  |
| N1 | 42 (19.0%) | 16 (17.4%) | 26 (28.0%) |  |
| N2 | 38 (14.0%) | 7 (7.6%) | 31 (33.3%) |  |
| N3 | 21 (13.2%) | 7 (7.6%) | 14 (15.1%) |  |
| Pathological stage |  |  |  | < 0.001†* |
| I | 58 (10.7%) | 52 (56.5%) | 6 (6.5%) |  |
| II | 57 (24.8%) | 29 (31.5%) | 28 (30.1%) |  |
| III | 65 (2.5%) | 10 (10.9%) | 55 (59.1%) |  |
| IV | 5 (4.1%) | 1 (1.1%) | 4 (4.3%) |  |
| αSMA |  |  |  | < 0.001§* |
| Median (IQR) | 8.99 (4.03–16.17) | 4.03 (2.50–6.86) | 15.75 (10.89–20.48) |  |

**(continued)**

| Tumor-infiltrating lymphocytes | |  |  |  |
| --- | --- | --- | --- | --- |
| CD8+ |  |  |  | < 0.001§* |
| Median (IQR) | 32.75 (17.50–57.00) | 42.63 (26.00–69.69) | 21.50 (11.88–46.50) |  |
| FoxP3+ |  |  |  | < 0.001§* |
| Median (IQR) | 16.33 (8.00–28.50) | 9.63 (3.81–16.25) | 24.25 (16.54–36.00) |  |
| Tumor-associated macrophages | |  |  |  |
| Iba1+ |  |  |  | 0.335§ |
| Median (IQR) | 238.50 (197.88–310.13) | 237.25 '193.69–294.63) | 248.50 (201.25–314.38) |  |
| CD163+ |  |  |  | < 0.001§* |
| Median (IQR) | 208.50 (116.00–284.38) | 116 (74.31–218.06) | 252.75 (206.75–321.75) |  |

Student’s *t*-test: §, Fisher’s exact test: †, Statistical significance: *P* < 0.05, IQR: interquartile range, SCC: squamous cell carcinoma

**Table S3. Results of univariate and multivariate Cox regression analyses of factors associated with disease-free survival among esophageal cancer patients.**

|  |  | **Univariate analysis** | | | **Multivariate analysis** | | |
| --- | --- | --- | --- | --- | --- | --- | --- |
| **Variable** | **Unfavorable/Favorable** | **HR** | **95%CI** | ***P* value** | **HR** | **95%CI** | ***P* value** |
| Histological type | Adenocarcinoma/SCC | 0.62 | 0.29–1.34 | 0.227 |  |  |  |
| Pathological T stage | T3-4/T1-2 | 4.09 | 2.73–6.13 | < 0.001* | 2.61 | 1.57–4.33 | < 0.001* |
| Pathological N stage | N1-3/N0 | 2.51 | 1.67–3.76 | < 0.001* |  |  |  |
| Neoadjuvant therapy | yes/no | 2.66 | 1.76–4.03 | 0.001* | 1.88 | 1.22–2.88 | 0.004* |
| αSMA | high/low | 2.64 | 1.77–3.93 | < 0.001* |  |  |  |
| IL-6 | high/low | 3.29 | 2.19–4.96 | < 0.001* | 1.76 | 1.06–2.92 | 0.028* |
| Tumor-infiltrating lymphocytes | |  |  |  |  |  |  |
| CD8+ | high/low | 0.62 | 0.42–0.91 | 0.014* |  |  |  |
| FoxP3+ | high/low | 2.46 | 1.65–3.67 | < 0.001* |  |  |  |
| Tumor-associated macrophages | |  |  |  |  |  |  |
| Iba1+ | high/low | 1.23 | 0.84–1.80 | 0.286 |  |  |  |
| CD163+ | high/low | 1.66 | 1.13–2.43 | 0.010* |  |  |  |

Cox proportional hazard model, Statistical significance: P < 0.05, HR: hazard ratio, CI: confidence interval, SCC: squamous cell carcinoma, SMA: smooth muscle actin, IL-6: interleukin-6, FoxP3: forkhead box p3, Iba1: ionized calcium-binding adaptor protein1
